# Application of Antimicrobial Peptides of the Innate Immune System in Combination With Conventional Antibiotics—A Novel Way to Combat Antibiotic Resistance?

**DOI:** 10.3389/fcimb.2019.00128

**Published:** 2019-04-30

**Authors:** Maria S. Zharkova, Dmitriy S. Orlov, Olga Yu. Golubeva, Oleg B. Chakchir, Igor E. Eliseev, Tatyana M. Grinchuk, Olga V. Shamova

**Affiliations:** ^1^Laboratory of Design and Synthesis of Biologically Active Peptides, Department of General Pathology and Pathophysiology, FSBSI Institute of Experimental Medicine, Saint Petersburg, Russia; ^2^Laboratory of Nanostructures Research, Institute of Silicate Chemistry of the Russian Academy of Sciences, Saint Petersburg, Russia; ^3^Nanobiotechnology Laboratory, Saint Petersburg National Research Academic University of the Russian Academy of Science, Saint Petersburg, Russia; ^4^Laboratory of Intracellular Signaling, Institute of Cytology of the Russian Academy of Science, Saint Petersburg, Russia

**Keywords:** antimicrobial peptides, synergy, antibiotics, drug-resistant bacteria, antibacterial activity

## Abstract

Rapidly growing resistance of pathogenic bacteria to conventional antibiotics leads to inefficiency of traditional approaches of countering infections and determines the urgent need for a search of fundamentally new anti-infective drugs. Antimicrobial peptides (AMPs) of the innate immune system are promising candidates for a role of such novel antibiotics. However, some cytotoxicity of AMPs toward host cells limits their active implementation in medicine and forces attempts to design numerous structural analogs of the peptides with optimized properties. An alternative route for the successful AMPs introduction may be their usage in combination with conventional antibiotics. Synergistic antibacterial effects have been reported for a number of such combinations, however, the molecular mechanisms of the synergy remain poorly understood and little is known whether AMPs cytotoxicy for the host cells increases upon their application with antibiotics. Our study is directed to examination of a combined action of natural AMPs with different structure and mode of action (porcine protegrin 1, caprine bactenecin ChBac3.4, human alpha- and beta-defensins (HNP-1, HNP-4, hBD-2, hBD-3), human cathelicidin LL-37), and egg white lysozyme with varied antibiotic agents (gentamicin, ofloxacin, oxacillin, rifampicin, polymyxin B, silver nanoparticles) toward selected bacteria, including drug-sensitive and drug-resistant strains, as well as toward some mammalian cells (human erythrocytes, PBMC, neutrophils, murine peritoneal macrophages and Ehrlich ascites carcinoma cells). Using “checkerboard titrations” for fractional inhibitory concentration indexes evaluation, it was found that synergy in antibacterial action mainly occurs between highly membrane-active AMPs (e.g., protegrin 1, hBD-3) and antibiotics with intracellular targets (e.g., gentamicin, rifampcin), suggesting bioavailability increase as the main model of such interaction. In some combinations modulation of dynamics of AMP-bacterial membrane interaction in presence of the antibiotic was also shown. Cytotoxic effects of the same combinations toward normal eukaryotic cells were rarely synergistic. The obtained data approve that combined application of antimicrobial peptides with antibiotics or other antimicrobials is a promising strategy for further development of new approach for combating antibiotic-resistant bacteria by usage of AMP-based therapeutics. Revealing the conventional antibiotics that increase the activity of human endogenous AMPs against particular pathogens is also important for cure strategies elaboration.

## Introduction

The XX century was marked by the undeniable success in the field of treatment and prophylactics of the infectious diseases. However, the spread of the drug resistance amongst pathogenic microbes poses a serious threat to the existing medical doctrine founded on the effective use of antibiotics (Rossolini et al., 2014; Ventola, 2015a). Said phenomenon endangers not only the successful cure of the infections caused by the resistant pathogens *per se*, but the whole spectrum of therapeutic procedures associated with the risk of infectious complications, including surgery, chemotherapy, etc. (Ventola, 2015a). The gravity of the problem at hand is publicly acknowledged worldwide. Thus, in 2015 the Global Action Plan on antimicrobial resistance (World Health Organization, 2015) was endorsed at the Sixty-eighth World Health Assembly. Necessary measures which must be taken in the face of the growing danger of antimicrobial resistance include different scientific, social, and economic aspects, but the development of new compounds or non-traditional methods effective against multidrug-resistant microorganisms is still the cornerstone of the whole strategy (World Health Organization, 2015).

Antimicrobial peptides (AMPs), evolutionary ancient and conservative tools of the innate immune system providing immediate response to the large set of various pathogens (Wiesner and Vilcinskas, 2010), are seen as promising candidates for the development of novel antibiotics (Gordon et al., 2005; Guaní-Guerra et al., 2010; Mahlapuu et al., 2016). These peptides are stored in granules of phagocytic cells and exert their effects in phagolysosomes or being secreted extracellularly; they are also widely expressed and released at epithelial surfaces and in a site of inflammation (Zasloff, 2002; Tosi, 2005; Maróti et al., 2011; Gallo and Hooper, 2012). AMPs remarkably differ in amino acid sequence and structure, but most of them are cationic and they can adopt an amphipathic conformation, thus, they are able to easily interact with the negatively charged components on the surface of bacterial cells and integrate into the lipid bilayers (Phoenix et al., 2013; Haney et al., 2017). The main mechanism of antibacterial action of AMPs is related with their ability to alter membrane permeability and damage its structure (Hancock and Rozek, 2002; Teixeira et al., 2012). This process is accompanied by the leakage of vital components, ions, and metabolites. Membrane destabilization additionally affects functioning of membrane-associated protein complexes (Nguyen et al., 2011; Wilmes et al., 2011). Some AMPs are non-membranolytic and penetrate bacterial membranes without disturbing their integrity. They have intracellular targets and interfere with the metabolic processes, including synthesis of the vitally important cell components (Brogden, 2005; Hale and Hancock, 2007; Le et al., 2017). Wide-scale multitargeted action is believed to be one of the reasons for the effectiveness of AMPs toward multidrug-resistant bacterial strains and an obstacle for the development of a high resistance level to such compounds (Wimley, 2010; Guilhelmelli et al., 2013; LaRock and Nizet, 2015). Overall, the beneficial features of AMPs are their broad spectrum of activity, swift, and effective bacterial killing that also complicates the resistance development, and possible additional effects such as immunomodulation (Mansour et al., 2014) and wound healing promotion (Ramanathan et al., 2002; Carretero et al., 2008; Pfalzgraff et al., 2018) demonstrated for certain peptides.

Combined use of antimicrobials, in particular those with different targets, is a known strategy to overcome multiple drug resistance (Pillai et al., 2005; Zimmerman et al., 2007; Turnidge, 2014; Ventola, 2015b). It also allows reducing the dosages, attenuating side effects and enhancing selectivity of compounds (Chou, 2006). The concept of combined use comes even more naturally concerning the AMPs, as in many tissues these compounds are expressed together in certain groups and are shown to possess synergy with each other (Chen et al., 2005; Dale and Fredericks, 2005; Lai and Gallo, 2009; Marxer et al., 2016). Such synergy is believed to be one of the keys to their successfulness in combating different resistance mechanisms invented by pathogenic bacteria during the long evolutional coexistence with the immune system of the host (Guilhelmelli et al., 2013; LaRock and Nizet, 2015). So, the combined use of AMPs with other antimicrobials such as conventional antibiotics definitely has potential to increase the effectiveness of both groups of compounds.

It is also noted that the high efficacy of individual antimicrobial agent *in vivo* may be in fact caused by a synergistic interaction with the antimicrobial peptides of the organism (Knappe et al., 2016). And vice versa, it is of interest to identify drugs that can enhance the effects of the body's own defense system (Sakoulas et al., 2014). Revealing such dependencies for human AMPs and antibiotics used in clinics may be of help for the optimization of cure strategies in some cases.

One of the prominent examples of the AMPs with distinctly membranolytic mode of action is protegrin 1 (PG-1) (Steinberg et al., 1997; Bolintineanu and Kaznessis, 2011), isolated from pig leukocytes (Kokryakov et al., 1993). It possesses a β-hairpin conformation stabilized by two disulfide bonds (Aumelas et al., 1996). In contrast, proline-rich AMPs are known for their low damaging action on membranes. A peculiar representative of this group is caprine bactenecin with the molecular weight of 3.4 kDa (ChBac3.4) from the leukocytes of the domestic goat *Capra hircus*, which exhibits a pronounced effect not only on Gram-negative, but also on Gram-positive bacteria, unlike the majority of proline-rich peptides (Shamova et al., 2009). This linear AMP, supposedly, has a dual mechanism of action, similar to the one described for Bac7 (1–35) (Mattiuzzo et al., 2007; Shamova et al., 2009). It is suggested that in low concentrations it acts by a non-lytic mechanism via intracellular targets, possibly, binding to the aminoacyl site of bacterial ribosomes (Krizsan et al., 2015; Roy et al., 2015) or interfering with the chaperone-dependent protein folding (Otvos, 2002; Scocchi et al., 2009; Zahn et al., 2013), as described for other proline-rich peptides. In higher concentrations ChBac3.4 actively damage the bacterial membranes, although not as fast as PG-1 (Shamova et al., 2009).

The main groups of AMPs presented in humans include cathelicidins and α- and β-defensins. The only human cathelicidin LL-37, when it contact with the bacterial membrane, adopts the conformation of an amphipathic α-helix (Vandamme et al., 2012). Defensins are stabilized by three disulfide bonds and are folded into the three-stranded antiparallel β-sheet (Lehrer and Lu, 2012). β-Defensins have an additional α-helical region at the N-terminus, which presumably facilitates anchoring of the peptide into bacterial membrane (Taylor et al., 2008; Machado and Ottolini, 2015). Antimicrobial activity of LL-37 and defensins is associated with the damaging of bacterial membranes, but it is noted that disruptive action of α-defensins on them progresses rather slowly, but is accompanied by the decrease in the synthesis of bacterial DNA, RNA and proteins, and the bacterium also loses an ability to form colonies (Lehrer and Lu, 2012). Solid-state NMR spectroscopy data support a “dimer pore” topology for the pores formed by α-defensin HNP-1 in model membranes (Zhang et al., 2011). These pores do not cause a significant disorder in the membrane lipid packing (Lehrer and Lu, 2012). Recent findings shows, that hBD-3 and HNP-1 can also inhibit the bacterial cell wall synthesis by specifically binding lipid II (de Leeuw et al., 2010; Sass et al., 2010).

Some AMP-like properties are also found in antimicrobial proteins, for example in lysozyme, which is widely presented in animals. It is an important component of the secretions of many glands, including mammary, salivary, and lacrimal, and of the mucus of nasopharynx and gastrointestinal tract (Fleming, 1922; Fábián et al., 2012). Lysozyme shows activity primarily against Gram-positive bacteria, catalyzing the lytic degradation of peptidoglycan, which is the major component of their cell wall (Masschalck and Michiels, 2003). However, there are reports suggesting that it can also act by a non-enzymatic mechanism probably similar to that of AMPs (Laible and Germaine, 1985; Ibrahim et al., 2001).

Unlike AMPs, clinically used antibiotics usually act via specific interactions with their molecular targets. Antibiotics can be classified on several groups depending on the vital process or structure of bacterial cell they affect. Due to the diversity of mechanisms of action they represent a perspective pool for searching synergistic interactions. In current study oxacillin, polymyxin B, gentamicin, amikacin, rifampicin, ofloxacin, erythromycin and meropenem were used. Oxacillin is a penicillinase-resistant β-lactam, which inhibits bacterial cell wall biosynthesis (Kong et al., 2010). Polymyxin B is a peptide antibiotic of bacterial origin, which increases the permeability of the bacterial membranes, similarly to AMPs (Carmona-Ribeiro and de Melo Carrasco, 2014). Gentamicin and amikacin are aminoglycosides and inhibit protein biosynthesis by the covalent irreversible binding to the 30S subunit of bacterial ribosomes (Davis, 1987). Rifampicin creates obstacles to the process of transcription by inhibiting bacterial DNA-dependent RNA polymerase (Wehrli, 1983). Ofloxacin is a quinolone antibiotic. It inhibits DNA-gyrase essential for DNA replication, thus, interfering with the bacterial cell division (Aldred et al., 2014). Erythromycin is a macrolide antibiotic. It reversibly binds to the 50S subunit of the ribosome and has a bacteriostatic effect (Keskar and Jugade, 2015). Meropenem is one of the carbapenems, β-lactam antibiotics, resistant to the majority of the bacterial β-lactamases (Papp-Wallace et al., 2011). The spread of resistance to carbapenems in recent years is one of the most concerning tendencies. Carbapenem-resistant strains, especially Gram-negative ones, including *Acinetobacter baumannii, Pseudomonas aeruginosa*, and various species of the *Enterobacteriaceae* family (in particular, *Klebsiella, Escherichia coli, Serratia*, and *Proteus*), are topping the global priority list of antibiotic-resistant bacteria, composed by World Health Organization (2017).

Another class of antimicrobial substances, which attracts the attention of researchers as a promising and effective tool against drug-resistant pathogens, is nanoparticles of various metals, in particular, of silver (Rai et al., 2012; Markowska et al., 2013; Cavassin et al., 2015), which has been known for its bactericidal properties for thousands of years (Alexander, 2009; Yang et al., 2018). The mechanism of antimicrobial action of silver nanoparticles has not been completely deciphered yet, but it is associated with their ability to affect the proper functioning of membrane associated proteins, including those of respiratory chain, stimulate ROS production and induce oxidative stress (Durán et al., 2010; Lara et al., 2011). The toxicity of nanoparticles was found to be much lower than that of ionic silver, however the problem of their side effects has not been completely solved (Durán et al., 2010, 2015; Stensberg et al., 2011). Reducing their effective concentrations by combined use with other antimicrobials can be beneficial.

The present study is dedicated to examining a combined action of natural antimicrobial peptides of different structure and mode of action [porcine PG-1, caprine bactenecin ChBac3.4, human α- and β-defensins (HNP-1, HNP-4; hBD-2, hBD-3), human cathelicidin LL-37] and egg white lysozyme with antibiotic agents, possessing varied targets in bacterial cells (selected antibiotics, silver nanoparticles), in a search of novel approaches of combined antimicrobial therapy for combating drug-resistant microbes. The tasks of the study included exploration of the activity of these combinations against a number of drug-sensitive strains, as well as several multi-resistant isolates. Combined hemolytic activity and cytotoxic action toward mammalian cells were also considered to assess the possibility of reducing toxic effects and increase selectivity.

## Materials and Methods

### Materials

#### Antimicrobial Peptides and Proteins

Chemically synthesized bactenecin ChBac3.4 was kindly provided by Dr. A. Kolobov (State Research Institute of Highly Pure Biopreparations, Saint Petersburg, Russia). It has been produced by Fmoc/tBu-strategy on solid-phase using a Liberty microwave peptide synthesizer (CEM Corp., USA), according to a standard synthesis protocols. Peptide purification was carried out by RP-HPLC (Gilson; France) on a Waters Prep-NovaPak 6 μm C18 (19 × 300 mm) column. Purity, as assessed by reverse phase analytical chromatography on DeltaPak 5 μm C18 100A (3.9 × 150 mm) column, was about 99%. The molecular mass was confirmed by MALDI-TOF MS. Protegrin 1 (PG-1) was a gift from Prof. R. Lehrer (University of California, Los Angeles). This peptide was produced by SynPep Corporation (USA); purity of synthetic PG-1 was 99 %. LL-37, human α- and β-defensins were purchased at Peptide Institute, Inc., Japan. Egg white lysozyme was obtained from BioChemica, Germany. We prepared stock solutions of the peptides from Peptide Institute, Inc., Japan, according to the instruction provided by the manufacturer. For other peptides the concentration of stock solutions was spectrophotometrically verified based on their molar extinction coefficients at 280 nm (Pace et al., 1995).

#### Silver Nanoparticles

Silver nanoparticles were synthesized by the photo-reduction of AgNO_3_ in presence of gelatin, which was used as an additional stabilizer. AgNO_3_ (99.9%, Chimmedsynthesis, Russia) and gelatin (Acros, Russia) were used for synthesis. 0.05 g of gelatin was added to 4 ml of H_2_O. Aqueous AgNO_3_ (6 ml, 1 M) was added to gelatin solution by continuous stirring. The solution obtained was placed into UV-reactor for UV irradiation for 24 h at room temperature. Dialysis was performed to remove unbound gelatin and ionic silver, and the low level of the latter in the samples was confirmed by the ionometry. The nanoparticles diameter (including gelatin coating) determined by electron microscopy was approximately 50 nm.

#### Antibiotics, Culture Media, and Other Reagents

All antibiotic powders, as well as phosphate-buffered saline (PBS) tablets, bovine serum albumin (BSA), o-nitrophenyl-β-D-galactoside (ONPG), ethylenediaminetetraacetic acid (EDTA), and methylthiazolyldiphenyl-tetrazolium bromide (MTT) were bought from Sigma, USA. Müller-Hinton broth M391 was purchased from Oxoid, Germany. Other reagents and media were supplied from BioloT, Russia.

#### Bacterial Strains

*Escherichia coli* ML-35p and MRSA (methicillin-resistant *Staphylococcus aureus*) ATCC 33591 bacterial strains were kindly provided by Professor R. Lehrer, University of California, Los Angeles, USA. *Micrococcus luteus* CIP A270 was obtained from the collection of the Department of Molecular Microbiology of the Institute of Experimental Medicine, Saint Petersburg, Russia. Antibiotic-resistant Gram-negative bacteria *Escherichia coli* ESBL 521/17 (resistant to ampicillin, amoxicillin/clavulonic acid, cefotaxime, ceftazidime, cefixime, aztreonam, netilmicin, ciprofloxacin, trimethoprim/sulfamethoxazole), *Pseudomonas aeruginosa* MDR 522/17 (resistant to meropenem, ceftazidime, cefixime, amikacin, gentamycin, netilmicin, ciprofloxacin, colistin), *Klebsiella pneumoniae* ESBL 344/17 (resistant to ampicillin), *Acinetobacter baumannii* 7226/16 (resistant to imipenem, gentamicin, tobramycin, ciprofloxacin, trimethoprim/sulfamethoxazole) and the susceptible strain of *Acinetobacter baumannii* were isolated from infected wounds, or from patient's urine in case of *E. coli* and generously provided by Prof. G.E. Afinogenov from Saint Petersburg State University and Dr. A. Afinogenova from the Research Institute of Epidemiology and Microbiology named after L. Pasteur, Saint Petersburg, Russia. Multidrug-resistant Gram-positive clinical isolate *Staphylococcus aureus* 1399/17 (resistant to ampicillin, oxacillin, gentamicin, amikacin, ofloxacin) originated from the same source.

#### Eukaryotic Cells

Human erythrocytes, mononuclear cells (PBMC) and neutrophils used in the study were isolated from peripheral blood of healthy donors.

To obtain erythrocytes, heparinized blood was centrifuged for 10 min at 300 g and 4°C. The supernatant liquid was removed, 10 ml of cooled PBS containing 4 mM of EDTA (pH 7.4) was added to the precipitate, and the mixture was centrifuged for 10 min under the same conditions. Then, red blood cells were washed three more times with cooled PBS. The sedimented red blood cells were used for the hemolytic test.

To obtain neutrophils and mononuclear cells, whole heparinized blood was diluted with sterile PBS in a 1:1 ratio and carefully inserted into the sterile polystyrene 50 ml tube upon the 5 ml of the sterile Ficoll-400 (Pharmacia, Sweden) with a density of 1.077. The tube was centrifuged at 600 g and 4°C for 40 min. After separation, the mononuclear “ring” located between the blood plasma and Ficoll was collected with a Pasteur pipette, transferred to another tube, and washed twice with 10 ml of sterile PBS by centrifugation for 10 min at 300 g and 4°C.

The pellet formed under the Ficoll-400 layer after separation contained both neutrophils and erythrocytes. It was also transferred into a separate tube, where the red blood cells were lysed with 0.83% ammonium chloride solution, which was added in a ratio of 1:4. After the thorough mixing, the suspension was incubated for 10 min at room temperature, and then centrifuged for 10 min at 300 g and 4°C. The resulting precipitate containing neutrophils was washed twice with the sterile PBS.

After washing, both mononuclear cells and neutrophils were resuspended in 1–2 ml of RPMI-1640 culture medium and immediately used in MTT test.

Peritoneal macrophages and Ehrlich ascites carcinoma (EAC) cells were obtained from (C57BL/6J × CBA/J) F1 hybrid male mice, 3 months old (body weight of 18–22 g). The animals were maintained under the standard vivarium conditions at room temperature with a 12-h light/12-h dark cycle, free access to food and water, according to the standards of laboratory animal welfare.

Before the cells extraction, mice were sacrificed by cervical dislocation in accordance with the supplement to the Directive 86/609/EEC and its update Directive 2010/63/EU addressing the recommendations for euthanasia of experimental animals (Close et al., 1996). To obtain peritoneal macrophages, healthy mice were used. The abdominal cavity of each animal was washed with 3 ml of sterile PBS using a Pasteur pipette. The culture of EAC cells was kindly provided by Dr. E.P. Kiseleva (Immunology Department of the Institute of Experimental Medicine, Saint Petersburg, Russia). On the tenth day after the intraperitoneal tumor inoculation, 0.5–1.5 ml of ascitic fluid containing EAC cells was obtained from the abdominal cavity of the animals. Murine cells (normal or tumor) were washed twice with 10 ml of sterile PBS and resuspended in RPMI-1640 medium as described above for human neutrophils and PBMC.

The human erythromyeloid leukemia cell line K562 was obtained from the collection of the Institute of Cytology RAS (Saint Petersburg, Russia) and cultured in RPMI-1640 medium containing gentamicin (100 μg/ml) and 10% of fetal bovine serum at 37°C, 5% CO_2_. Doxorubicin-resistant K562 cells were obtained from the parent line by continuous exposure of the cells to increasing concentrations of doxorubicin from 0.00016 to 2 μg/ml as described (Grinchuk et al., 1998), and their resistance to doxorubicin was 1–2 orders of magnitude higher than in the original K562 cells. Drug resistance was maintained by monthly selection with the antibiotic (2 μg/ml in the culturing medium). Doxorubicin was removed from the medium 3 days prior to beginning any experiments. Before the testing K562 cells were washed twice with PBS and resuspended in RPMI-1640 in the same way as the other cell types.

### Antibacterial Assays

#### Broth Microdilution Assay

Prior to the “checkerboard” synergy test the minimal inhibitory concentrations (MIC) of individual substances were determined using broth microdilution assay. Testing was performed in 60-well polystyrene Terasaki microplates with V-shaped bottom generally according to the guidelines of the European Committee for Antimicrobial Susceptibility Testing with subtle modifications designed for AMP testing (Tossi et al., 1997; Wiegand et al., 2008). Cultivation of bacteria was carried out in 2.1% Müller-Hinton broth at 37°C with shaking. Two-fold serial dilutions of peptides, antibiotics and other antimicrobials were made with 10 mM sodium phosphate buffer, pH 7.4, containing 0.1% of BSA. The plates were preliminary incubated with 0.1% BSA for 1 h at 37°C to reduce a non-specific binding of peptides to the plates. After that BSA was removed, and serial dilutions were added into the plate wells in triplicates followed by the same volume of the suspension of bacterial cells in logarithmic phase of growth in concentration of 1 × 10^6^ CFU/ml. After the 18 h overnight incubation at 37°C, the MIC value for each compound was determined as its lowest concentration completely inhibiting the visible growth of bacteria. The final results were calculated as median values based on 3–6 independent experiments.

#### Checkerboard Titration Method

The combined antimicrobial action of AMPs and other antimicrobial compounds was assessed using checkerboard titration approach under the conditions similar to that of the microdilution assay. According to checkerboard titration template, one component (A) of the combination was diluted along the rows of the plate, while the other (B) was diluted down the columns, thereby creating the variety of mixtures with different concentrations of tested compounds. 2.5 μl of corresponding solutions of the components A and B were added into each well of the plate. Into the first column and the last row the same amount of 10 mM sodium phosphate buffer, pH 7.4, containing 0.1% of BSA was introduced instead of the second component. Thus, all wells contained 5 μl of antimicrobial compounds solutions (solo or in combination). The same volume of bacterial suspension, prepared as described for microdilution assay, was added into each well. The results were recorded after the overnight incubation, visually indicating the presence or absence of the microbial growth in the corresponding wells. Based on the obtained data the isobolograms of combined action were plotted and/or Fractional Inhibitory Concentration Indices (FICI) were calculated. The latter was done as follows: FIC Index = [A]/[MIC A] + [B]/[MIC B], where [A] and [B] are respective concentrations of substances A and B in their combination effectively inhibiting bacterial growth, and [MIC A] and [MIC B] are the individual MICs of A and B when they are used alone. Depending on the minimal value of the FICI the combined effect of the combination was classified as antagonism (FICI > 2), independent action (1 < FICI ≤ 2), additivity (0.5 < FICI ≤ 1) or synergy (FICI ≤ 0.5), as is recommended in the literature and due to some drawbacks of the serial dilution method (Hsieh et al., 1993; Orhan et al., 2005; Fehri et al., 2007). FICIs were assessed in 3–6 independent experiments and median values were used to define the nature of interaction.

#### Bacterial Membrane Permeabilization Assay

The ability of antimicrobial peptides, alone and in combinations, to increase the permeability of the cytoplasmic membrane of Gram-negative bacteria was examined using a previously described procedure (Lehrer et al., 1988). The method is based on the certain features of the specifically designed *E. coli* ML-35p bacterial strain. As its parental strain *E. coli* ML-35, this bacterium constitutively expresses cytoplasmic β-galactosidase, but is unable to transport β-galactoside containing substrates through the inner membrane in normal circumstances due to the lack of lactose permease. In addition, *E. coli* ML-35p also possesses periplasmic β-lactamase. If the cytoplasmic membrane of the bacterium is damaged, the chromogenic marker o-nitrophenyl-β-D-galactoside (ONPG) penetrates into the cell and, when digested by bacterial β-galactosidase, produces a colored product o-nitrophenol. The accumulation of the latter can be spectrophotometrically detected at a wavelength of 420 nm.

*Escherichia coli* ML-35p was cultivated in the sterile 3% Trypticase soy broth (TSB) for 18 h at 37°C, then washed three times with 10 mM sodium phosphate buffer, pH 7.4 (10 min centrifugation at 600 g and 4°C), diluted up to the OD of 0.4 at 620 nm (1 × 10^8^ CFU/ml concentration), and used immediately or kept on ice until the start of the test. The incubation wells for the testing contained antimicrobial components (alone or in combinations) in concentrations equal to ¼ of MIC, 10 mM sodium phosphate buffer with 100 mM NaCl (pH 7.4), 2.5 mM ONPG, and 2.5 × 10^7^ CFU/ml of bacteria in a stationary growth phase washed out of the culture medium. Controls contained equivalent volume of acidified water instead of antimicrobial agent(s). The assay was started by adding the bacteria and was run at 37°C, with periodical shaking. Optical density (OD) measurement at 420 nm was performed every minute for 1–2 h using SpectraMax 250 Microplate Spectrophotometer (Molecular Devices, USA) and its SoftMax PRO software.

### Cytotoxicity Assays

#### Combined Hemolytic or Cytotoxic Action

Examining the combined cytotoxic effects of various combinations of AMP with other active compounds we applied the same principle based on the Loewe additivity paradigm (Greco et al., 1992) that was used in the analysis of the combined antibacterial action. We calculated Fractional Effective Concentration Index (FECI), which could be seen as a particular case of the interaction index (I) proposed by Berenbaum (1977) or of the similar combination index (CI) proposed by Chou and Talalay (1983), according to the equation below:

(1) $$\begin{array}{l} \text{FEC Index}=\left[\text{A}\right]/\left[\text{MEC A}\right]+\left[\text{B}\right]/\left[\text{MEC B}\right]. \end{array}$$

Here, [MEC A] and [MEC B] are the minimum effective concentrations (MEC) of substances A and B, when they are used alone. MEC is defined as the minimum concentration of the compound, at which the statistically significant difference with the control samples containing intact cells (0% hemolysis for hemolytic activity assay or 100% cell survival for MTT-test) is found, concerning the studied effect (thereby it is called “effective”). [A] and [B] are respective concentrations of substances A and B in their combination, where the same statistically significant difference is detected. A non-parametric Mann-Whitney *U*-test (*p* < 0.05 or *p* < 0.01) was used for the mentioned statistical comparison.

We have chosen the same intervals as that of FICI to determine synergy, additivity, independent action, and antagonism based on the minimal FECI values. However, in case of cytotoxic or hemolytic effect we were interested mainly in testing the possibility of additive or synergistic interaction itself, and not in assessing the exact magnitude of such interaction. Thus, in contrast to the “checkerboard titration” method used for combined antimicrobial activity investigation, studying the combined cytotoxic effect, we considered only two combinations of concentrations for the substances A and B in the mixture:

(½ MEC A + ½ MEC B), which, if found effective, provides the FECI value of 1 (the threshold value for additivity);
(¼ MEC A + ¼ MEC B), which, if found effective, provides the FECI value of 0.5 (the threshold value for synergetic interaction).

The combined action of the test substances was classified as independent (in the absence of statistically significant difference found for both combinations of concentrations), additive (if the statistically significant difference was shown only for a combination of ½ MEC A + ½ MEC B), or synergistic (if there is a statistically significant difference from the control samples for both combinations of concentrations). When action on tumor cells was tested in combination with antitumor antibiotic the lower concentrations up to (116 MEC A + 116 MEC B) as in checkerboard titration were additionally examined to assess the magnitude of synergistic effect more precisely. The final conclusion was made based on the three independent experiments.

#### Hemolysis Test

Hemolytic activity was investigated by incubating tested compounds in various concentrations and their combinations with a suspension of washed human red blood cells. Two hundred eighty microliters of erythrocyte precipitate obtained as described in section Eukaryotic Cells was added into the sterile 15 ml tube and its volume was adjusted to 10 ml with cooled PBS. Using 0.5 ml plastic tubes, 3 μl of the tested compound serially diluted in PBS or of the combination of two compounds were mixed with the 27 μl of the prepared erythrocyte suspension for each sample. For positive control (100% lysis of erythrocytes) 3 μl of the detergent (10% Triton X-100 solution in PBS) were added to the 27 μl of the erythrocyte suspension, and for negative control (0% lysis of erythrocytes) 3 μl of the PBS were used instead of any cell-damaging compound. Resulting 2.5% (v/v) erythrocyte suspensions were incubated for 30 min at 37°C with periodical shaking. After incubation, the hemolysis process was stopped by the addition of 75 μl of cooled PBS. The samples were centrifuged at 5,000 g and 4°C for 4 min. The optical density of the supernatants was measured at a wavelength of 540 nm using SpectraMax 250 Spectrophotometer (Molecular Devices, USA). The experiments were repeated three times. In each experiment all the test or control samples were made in triplicates. The percentage of hemolysis of erythrocytes was assessed by the following formula:

(2) $$\begin{array}{l} \text{Hemolysis}(\%)=({\text{OD}}_{\text{sample}}-{\text{OD}}_{0\%\text{lysis}})/({\text{OD}}_{100\%\text{lysis}}\\ \;-{\text{OD}}_{0\%\text{lysis}})\times 100\%, \end{array}$$

where OD_sample_, OD_0%lysis_, and OD_100%lysis_ are values of the OD at 540 nm for the test sample, negative (0% lysis) control, and positive (100% lysis) control, respectively. However, due to the design of the study of combined action, described above in Combined Hemolytic or Cytotoxic Action, the obtained OD_sample_ and OD_0%lysis_ values were rather used to determine MECs and FECI by the Mann-Whitney *U*-test (*p* < 0.05, *n*_1_, *n*_2_ = 3).

#### MTT-Test

Standard MTT-test (Mosmann, 1983) was used for the examination of the cytotoxic activity of antimicrobial compounds and their combinations toward varied eukaryotic cells. Briefly, cell suspensions in RPMI-1640 prepared as described in Eukaryotic Cells were dispensed into the 96-well microplates (2 × 10^5^ cells/well). Then, serial dilutions of AMPs, or other antibiotic compounds, or their mixtures in RPMI-1640 were added. In positive controls (100% cell survival) the according volume of RPMI-1640 without test substances was added instead. The final volume in each well was 100 μl. Negative controls (0% cell survival) were made with 100 μl of the sterile RPMI-1640. The plates were incubated at 37°C under 5% CO2 for 24 h. Four hours before the end of incubation, 10 μl of metabolic marker MTT−3-(4,5-dimethylthiazol-2-yl)-2,5-diphenyltetrazolium bromide (5 mg/ml, diluted in PBS) was added to each well. Incubation was stopped by adding 90 μl of isopropanol containing 0.04 M HCl. Content of the wells was thoroughly mixed, so that the formazan crystals, formed in case of the reduction of MTT in presence of actively metabolizing cells, were fully dissolved. The optical density of the samples was evaluated at 540 nm, subtracting the background absorbance at 690 nm, by SpectraMax 250 Spectrophotometer (Molecular Devices, USA). The experiments were repeated at least three times. In each experiment test samples were made in triplicates and control samples were made in hexaplicates. OD values of samples and of positive controls (100% cell survival) were statistically compared using the Mann-Whitney *U*-test (*p* < 0.05 or *p* < 0.01; *n*_1_ = 2–3, *n*_2_ = 5–6) to determine MECs and FECI (as previously defined). The % of surviving cells for test samples could be found as:

(3) $$\begin{array}{l} \text{Survival}(\%)=({\text{OD}}_{\text{sample}}-{\text{OD}}_{0\%\text{survival}})/({\text{OD}}_{100\%\text{survival}}\\ \;-{\text{OD}}_{0\%\text{survival}})\times 100\% \end{array}$$

where OD_sample_, OD_0%survival_, and OD_100%survival_ are values of the OD at 540 nm minus the OD at 690 nm for the test sample, negative (0% cell survival) control, and positive (100% cell survival) control, respectively.

#### Ethics Statement

In this study erythrocytes, mononuclear cells and neutrophils, used for evaluation of cytotoxic activity of the peptides and antibiotics, were obtained from blood of healthy donors. Since authors of this article served as such the donors, ethical approval was not required by the Ethics Committee of the Institute of Experimental Medicine. All subjects/authors gave their written consent.

## Results

### Effects on Bacterial Cells

#### Combined Antibacterial Action

At the first stage of the study we explored the combined action of AMPs and lysozyme with antibiotics against drug-susceptible bacteria. MIC values for individual substances are displayed in Table 1. The individual antimicrobial activity of oxacillin, HNP-4 and hBD-2 against *E. coli* ML-35p and that of LL-37 and HNP-4 against MRSA ATCC 33591 is low. The combinations including named substances were not further tested against the corresponding microorganisms after it was verified that an addition of ¼ MIC of the second antimicrobial to the ½ MIC of the component with low activity was not effective (data not shown). For lysozyme, which also is not active against these two bacteria, that, however, was not the case, thus, it was not excluded. The minimal FICIs for tested combinations of AMPs or lysozyme with antibiotics are given in Table 2. Values corresponding to synergistic effect (FICI ≤ 0.5) are shown in bold type.

**Table 1:** Antimicrobial activity of individual fractions of AMPs and antibiotics against Gram-negative and Gram-positive bacteria.

	**MIC^a^ (μM) toward bacterial strains**			
	**Gram-negative**		**Gram-positive**	
**Sample**	***E. coli* ML-35p**	***A. baumannii* (clin. isol.)**	**MRSA ATCC 33591**	***M. luteus* CIP *A270***
LL-37	10	2.5	50	1.25
HNP-1	50	0.6	2.5	0.1
HNP-4	>100 [200]	0.8	12.5	0.2
hBD-2	>100 [200]	6.25	>100 [200]	1.0
hBD-3	30	1.2	6.8	0.6
PG-1	3.1	1.6	3.8	0.5
ChBac3.4	3.1	1.6	7.2	1.6
Lysozyme	>250 [500]	1.2	>250 [500]	0.08
Oxacillin	>250 [500]	25	7.5	3.1
Polymyxin B	0.4	1.25	12.5	0.6
Gentamicin	0.625	0.06	0.16	0.16
Rifampicin	10	0.125	0.003	0.003
Ofloxacin	0.125	0.3	0.625	5

a *Minimal inhibitory concentrations (MIC) values are medians of 3–6 independent experiments made in triplicates. If actual MIC value was out of the tested concentrations range, it was assessed as twice the maximal tested concentration; the corresponding value is given in square brackets*.

**Table 2:** Antimicrobial activity of combinations of AMPs with antibiotics against Gram-negative and Gram-positive bacteria.

	**Minimal FICIs^a^ of AMP\antibiotic(AB) combinations against bacteria**										
	**Gram-negative**						**Gram-positive**				
**AMP**\**AB**	**RIF**	**PMB**	**GEN**	**OFL**	**OX**	**AMP**\**AB**	**RIF**	**PMB**	**GEN**	**OFL**	**OX**
	***E. coli*** **ML-35p**						**MRSA ATCC 33591**				
LL-37	0.75	0.75	0.75	1.12	–	HNP-4	0.75	1.12	1	1	1.12
HNP-1	0.56	1	0.62	1.12	–	HNP-1	**0.5**	1.12	0.75	1	1
hBD-3	0.75	0.75	**0.28**	0.53	–	hBD-3	**0.5**	1	0.75	0.75	0.75
ChBac3.4	**0.5**	1	1	0.75	–	ChBac3.4	0.75	0.62	0.75	1	**0.5**
PG-1	0.75	1	**0.38**	0.62	–	PG-1	0.75	0.62	1	1	0.75
Lysozyme	0.62	**0.25**	0.53	1.12	–	Lysozyme	0.62	0.56	0.53	1.12	0.53
	***A. baumannii*** **(clin.isol.)**						***M. luteus*** **CIP A270**				
LL-37	1.12	1	0.75	1	1.5	LL-37	1	0.56	**0.25**	0.75	0.75
HNP-1	0.75	1	0.62	0.75	0.75	HNP-1	0.75	0.75	**0.19**	1	1
HNP-4	1.12	1	0.62	1	1	HNP-4	1	0.75	0.56	1	0.75
hBD-2	**0.5**	**0.38**	0.75	1	1	hBD-2	0.75	**0.5**	**0.19**	0.75	0.75
hBD-3	1.12	0.62	**0.5**	1.12	1	hBD-3	0.62	0.75	**0.31**	0.62	1
ChBac3.4	0.75	0.75	1	1	0.75	ChBac3.4	0.75	0.75	0.62	**0.5**	0.75
PG-1	1.12	0.75	**0.38**	1	1.12	PG-1	0.75	0.62	**0.12**	1	0.75
Lysozyme	1	**0.5**	0.75	1	1	Lysozyme	0.75	0.62	0.62	0.62	0.75

*AMP, antimicrobial peptide; RIF, rifampicin; PMB, polymyxin B; GEN, gentamicin; OFL, ofloxacin; OX, oxacillin*.

a *Fractional Inhibitory Concentration Indices (FICI) values are medians of 3–4 independent experiments; FICI > 2 indicates antagonism, 1 < FICI ≤ 2 shows independent action, 0.5 < FICI ≤ 1 corresponds to additivity, FICI ≤ 0.5 denotes synergy; synergy cases are set off in bold type*.

**Figure 1:**
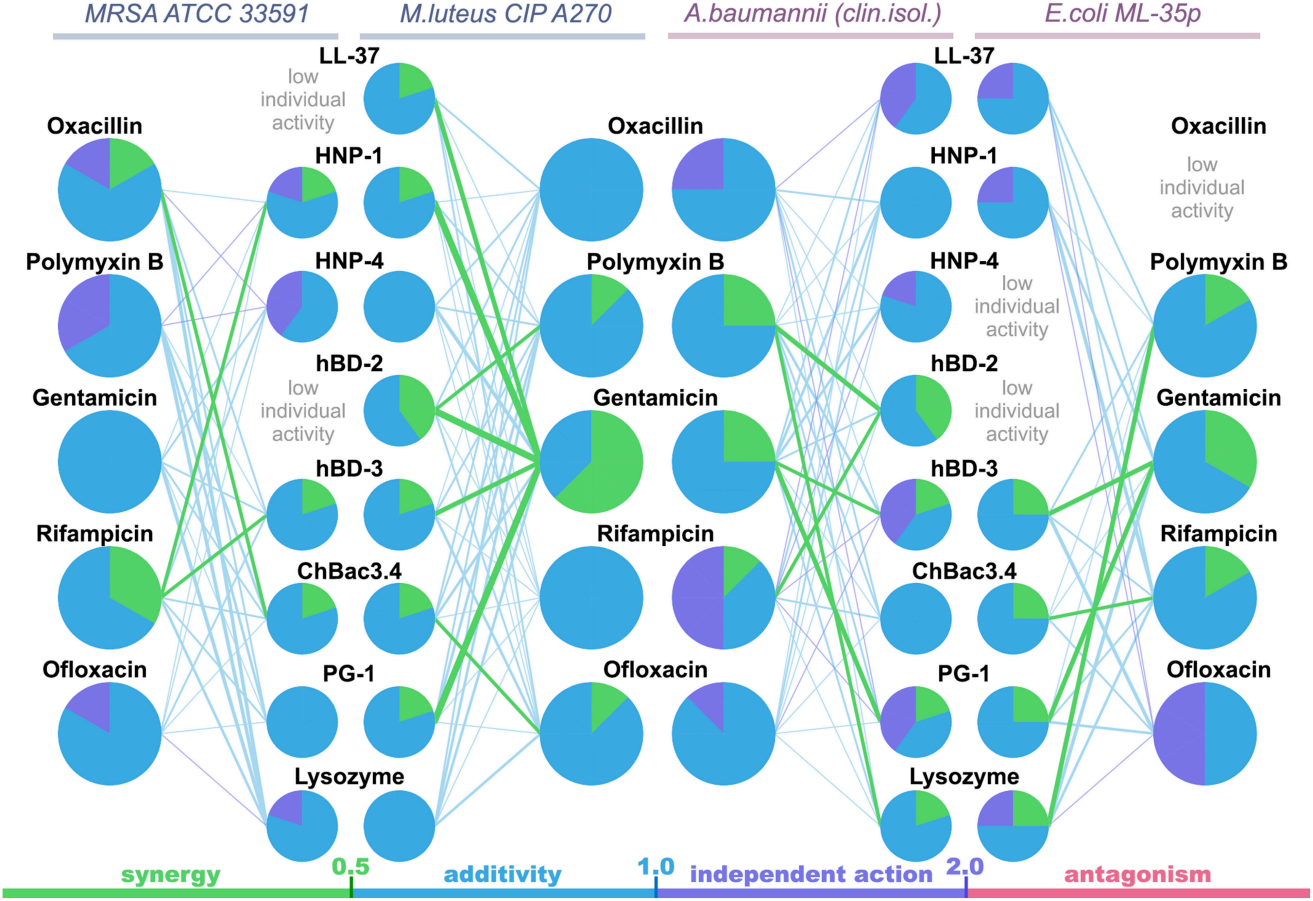
Polygonograms of combined antibacterial action of AMPs and conventional antibiotics shown in Table 2. Edges color and thickness reflect the type of interaction (green for synergy, blue for additivity, violet for independent action, red for antagonism) and the Fractional Inhibitory Concentration Index (FICI) value (the lesser is FICI, the thicker is the edge). Nodes contain pie charts showing the ratio of different interaction types for each antimicrobial peptide (AMP) or antibiotic in the particular polygonogram.

The data are also represented as polygonograms (Figure 1) to better visualize the general picture of interactions. Given polygonograms are in fact complete bipartite graphs, which can be considered as labeled or even weighted, as the minimal FICI value for the particular combination is coded by thickness (the lesser – the thicker) and color of the edge connecting the nodes representing the components of the said combination. Pie charts reflecting various types of interactions for the selected compounds are placed in the corresponding nodes as in Cokol et al. (2011). The color coding used for both charts and edges is: green for synergy (FICI ≤ 0.5), blue for additivity (0.5 <FICI ≤ 1), violet for independent action (1 < FICI ≤ 2) and red for antagonism (FICI > 2).

Synergistic effects are found for combinations of bactenencin ChBac3.4 with rifampicin against Gram-negative bacteria *E. coli* ML-35p; with oxacillin against Gram-positive bacteria MRSA ATCC 33591, which has a relatively high resistance to this antibiotic; with ofloxacin against Gram-positive bacteria *M. luteus* CIP A270. Membranolytic AMP PG-1 demonstrates synergistic action with gentamicin against both Gram-negative microorganisms tested, and, albeit to a lesser extent, against the Gram-positive strain *M. luteus* CIP A270.

The mutual enhancement of antimicrobial activity occurs in case of the combined use of lysozyme and polymyxin B on Gram-negative bacteria. The action of lysozyme with some antibiotics against bacteria, which are highly resistant to the former, is also quite close to synergistic: namely, with gentamicin against *E. coli* ML-35p and with gentamycin, oxacillin, and polymyxin B against MRSA ATCC 33591.

Amongst tested human AMPs, synergistic effect is detected in following combinations: hBD-3 with gentamicin against Gram-negative bacteria; hBD-2 with rifampicin and polymyxin B against susceptible *A. baumannii*, as well as with polymyxin B against *M. luteus* CIP A270; HNP-1 or hBD-3 with rifampicin against MRSA ATCC 33591; LL-37, or HNP-1, or hBD-2, or hBD-3 with gentamicin against *M. luteus* CIP A270. Interaction close to synergy is observed for combinations of HNP-1 with rifampicin and hBD-3 with ofloxacin against *E. coli* ML-35p, as well as for LL-37 with polymyxin B and HNP-4 with gentamicin against *M. luteus* CIP A270.

In total, counting the combined action of each AMP/antibiotic combination on each tested bacteria separately, additivity is observed in 75.4% of cases (101 of 134), and synergy is found in 14.2% of cases (19 of 134). However, from 40 examined combinations of AMPs and conventional antibiotics 13 (that is 32.5%) shows synergistic effect against at least one of the tested bacterial strains. Ten of this thirteen combinations include gentamicin, rifampicin or ofloxacin, which affect intracellular biosynthesis of proteins and nucleic acids. No antagonistic interactions are found.

**Figure 2:**
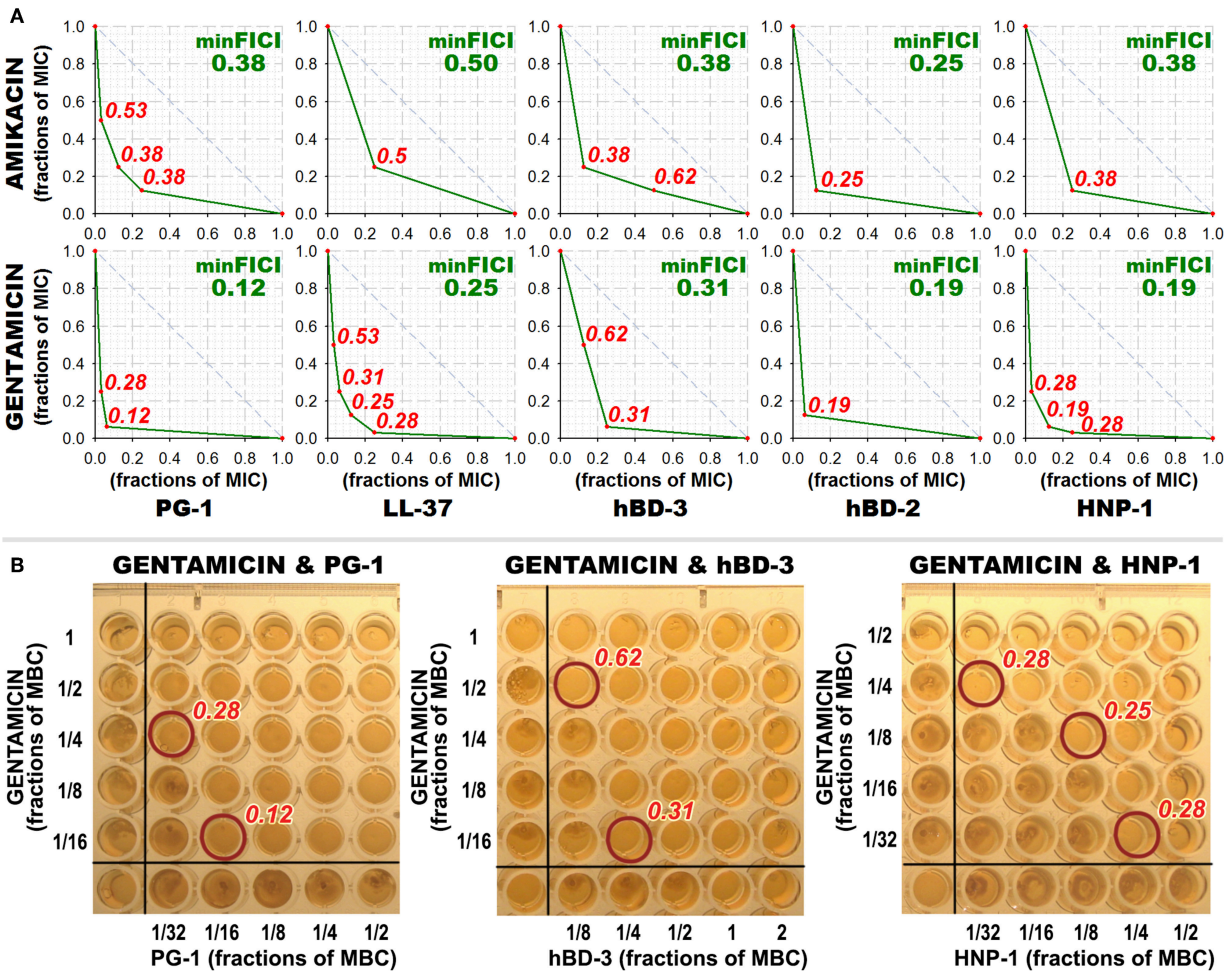
Combined antibacterial action of aminoglycosides amikacin and gentamicin with AMPs against *M. luteus* CIP A270. **(A)** Isobolograms demonstrating synergistic effect of combinations of amikacin and gentamicin with antimicrobial peptides (AMPs) against *M. luteus* CIP A270. Concentrations of components are given in fractions of their individual minimal inhibitory concentrations (MICs). Diagonal dashed line illustrates the model additive interaction expected for Loewe additivity criterion. Minimal FICI is shown in the upper right corner of each isobologram, its value is median of 3–4 independent experiments showing similar results; isobolograms are samples from one of these experiments. **(B)** Microbicidal action of combinations of amikacin and gentamicin with AMPs against *M. luteus* CIP A270. After the checkerboard titration test, wells content was diluted 10 times with phosphate-buffered saline, and 3 μl of the dilution was putted on tryptic soy agar placed in the wells of a 96-well plate using the same template; 24 h incubation at 37°C was performed and microbial growth was subsequently observed. Photographs show the typical samples of the results obtained.

Interestingly, most of the synergy cases occur in combinations of AMPs with gentamicin (the effect is revealed for LL-37, PG-1, HNP-1, hBD-2, hBD-3). After this finding, measurements of combined antimicrobial action with listed AMPs were replicated using another aminoglycoside antibiotic – amikacin. Activity was examined against *M. luteus* CIP A270, where all these combinations with gentamicin had previously shown synergy, and similar results with amikacin were obtained. Corresponding isobolograms are shown at Figure 2A. After the checkerboard titration test, the content of the wells was ten-fold diluted and putted onto the solid nutrient medium. Microbial growth was observed after a 24 h incubation at 37°C, showing that the effects of combinations were not just bacteriostatic, but indeed bactericidal (examples are shown at Figure 2B).

For PG-1 and ChBac3.4, which were most active amongst tested AMPs, combined antibacterial effects with antibiotics were further explored against drug-resistant clinical isolates. The MICs and minimal FICIs values are shown in Tables 3, 4, respectively. As none synergistic interactions were previously found for combinations of either PG-1 or ChBac3.4 with polymyxin B, the latter was excluded from the list of tested antibiotics. Meropenem and erythromycin were examined instead.

**Table 3:** Antimicrobial activity of individual fractions of AMPs and antibiotics against drug-resistant clinical isolates.

	**MIC^a^ (μM) against drug-resistant clinical isolates**				
	**Gram-negative**				**Gram-positive**
**Sample**	***E. coli* ESBL 521/17**	***A. baumannii* 7226/16**	***P. aeruginosa* MDR 522/17**	***K. pneumoniae* ESBL 344/17**	***S. aureus* 1399/17**
PG-1	0.4	6.25	1.6	1.6	1.0
ChBac3.4	6.25	12.5	6.25	6.25	12.5
Oxacillin	>50 [100]	>50 [100]	>50 [100]	>50 [100]	>50 [100]
Meropenem	0.04	>50 [100]	25	0.04	6.25
Erythromycin	>50 [100]	>50 [100]	>50 [100]	50	>50 [100]
Amikacin	1.0	6.25	1.0	0.25	>50 [100]
Ofloxacin	50	25	>50 [100]	0.25	>50 [100]

a *Minimal inhibitory concentrations (MIC) values are medians of 3–6 independent experiments made in triplicates. If actual MIC value was out of the tested concentrations range, it was assessed as twice the maximal tested concentration; the corresponding value is given in square brackets*.

**Table 4:** Antimicrobial activity of combinations of AMPs with antibiotics against drug-resistant clinical isolates.

	**Minimal FICIs^a^ of antibiotic(AB)\AMP combinations against drug-resistant clinical isolates**									
	**Gram-negative**								**Gram-positive**	
	***E. coli*** **ESBL 521/17**		***A. baumannii*** **7226/16**		***P. aeruginosa*** **MDR 522/17**		***K. pneumoniae*** **ESBL 344/17**		***S. aureus*** **1399/17**	
**AB\AMP**	**PG-1**	**ChBac3.4**	**PG-1**	**ChBac3.4**	**PG-1**	**ChBac3.4**	**PG-1**	**ChBac3.4**	**PG-1**	**ChBac3.4**
OX	1.12	1.12	0.75	1.12	1.12	1.12	1.12	1.12	1.12	0.25^*^
MEM	1.0	1.0	0.38^*^	0.5^*^	1.0	0.62	1.0	1.0	0.25^*^	0.31^*^
ERY	0.62	0.38^*^	1.12	0.75	0.5^*^	0.38^*^	0.25^*^	0.25^*^	0.75	0.5^*^
AMK	**0.38**	0.62	0.38^*^	0.5^*^	**0.5**	**0.5**	**0.31**	**0.38**	1.0	0.125^*^
OFL	0.75	0.75	0.62	0.5^*^	1.12	0.75	**0.5**	0.75	0.56	0.125^*^

*AMP, antimicrobial peptide; OX, oxacillin; MEM, meropenem; ERY, erythromycin; AMK, amikacin; OFL, ofloxacin*.

a *Fractional Inhibitory Concentration Indices (FICI) values are medians of 3–6 independent experiments; FICI > 2 indicates antagonism, 1 < FICI ≤ 2 shows independent action, 0.5 < FICI ≤ 1 corresponds to additivity, FICI ≤ 0.5 denotes synergy; synergy cases are set off in bold type*.

* *Bacterium has moderate or high resistance to the antibiotic being part of the synergistic combination*.

Individually PG-1 is active against all of the antibiotic-resistant isolates (Table 3) in the same concentrations as against laboratory drug-sensitive strains (Table 1); for ChBac3.4 the MICs are just slightly higher. For combinations of AMPs with aminoglycoside amikacin, synergy is found in almost all cases with the exception of the action against *S. aureus* 1399/17 for PG-1 and against *E. coli* ESBL 521/17 for ChBac3.4 Though only *S. aureus* 1399/17 shows high resistance to amikacin itself as it can be seen in Table 3. Interestingly, the cases of synergy with erythromycin, to which all clinical isolates possesses significant level of resistance, are also quite numerous. Rather similarly to amikacin, this antibiotic affects bacterial ribosomes, albeit binding to another subunit. The synergistic effect is found against *P. aeruginosa* MDR 522/17 and *K. pneumoniae* ESBL 344/17 for combination of the antibiotic with PG-1, and against all tested strains, except *A. baumannii* 7226/16, for combination with ChBac3.4. Combination of ofloxacin with PG-1 shows synergy only against *K. pneumoniae* ESBL 344/17, which is susceptible to this antibiotic. On the other hand, for such combination with ChBac3.4, synergistic action is detected against the ofloxacin-resistant strains *A. baumannii* 7226/16 and *S. aureus* 1399/17. The combined effect of both PG-1 and ChBac3.4 with meropenem is synergistic against *A. baumannii* 7226/16, which has a high level of resistance to this antibiotic, and against *S. aureus* 1399/17, for which MIC of meropenem is also higher than that for susceptible strains. The combination of ChBac3.4 and oxacillin, previously shown to be synergistic against the laboratory strain MRSA ATCC 33591, demonstrates similar effect against clinically isolated *S. aureus* 1399/17, which is highly resistant to oxacillin.

**Figure 3:**
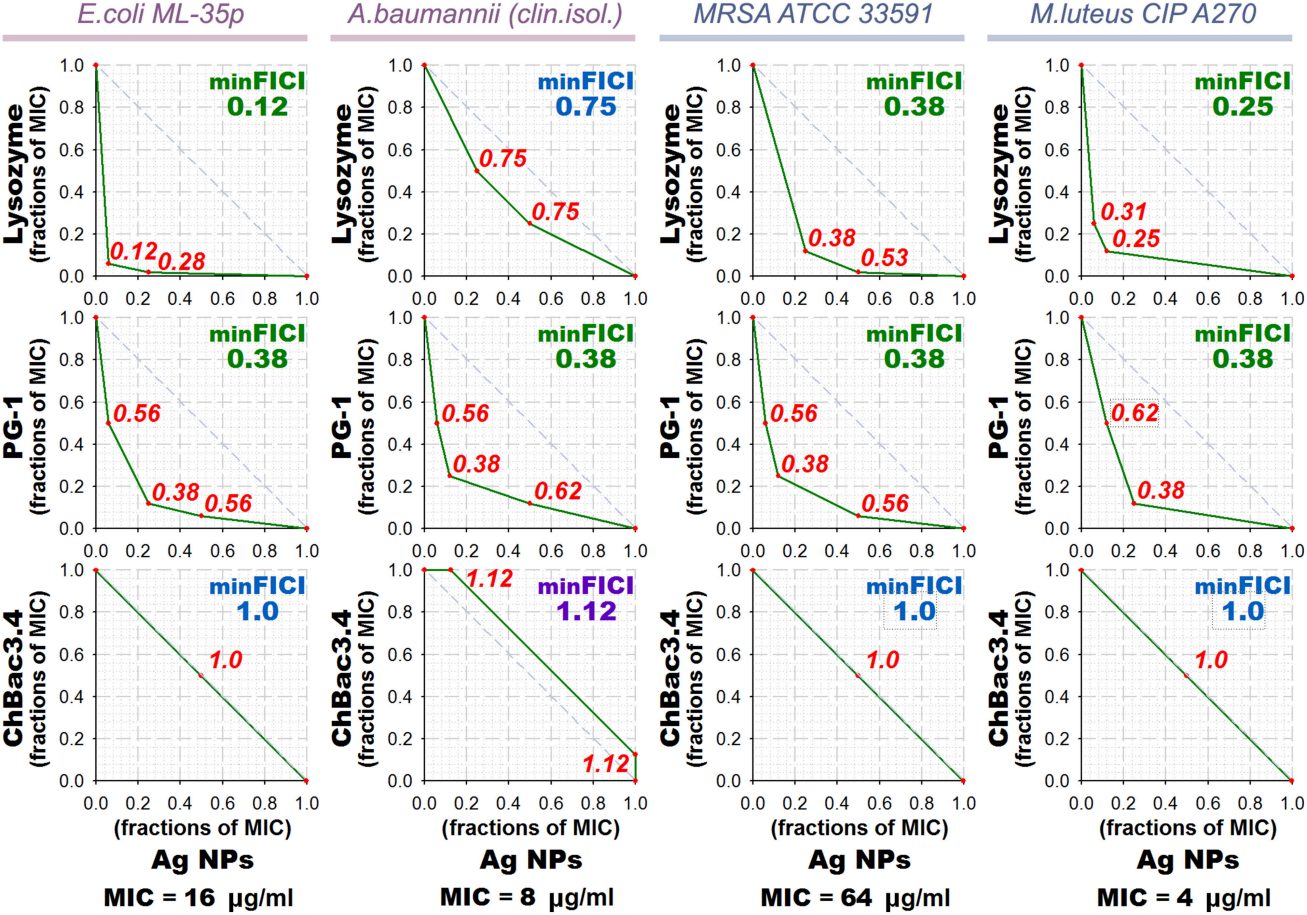
Isobolograms of combined antibacterial action of gelatin-coated silver nanoparticles (Ag NPs) with lysozyme and AMPs. Concentrations of components are given in fractions of their individual minimal inhibitory concentrations (MICs). MICs values for silver nanoparticles alone are shown under the graphs. Diagonal dashed line illustrates the model additive interaction expected for Loewe additivity criterion. Minimal Fractional Inhibitory Concentration Index (FICI) is shown in the upper right corner of each isobologram, its value is median of 3–4 independent experiments showing similar results; it is green for synergy (FICI ≤ 0.5), blue for additive effect (0.5 < FICI ≤ 1), and violet for independent action (1 < FICI ≤ 2). Isobolograms represent samples from one of the experiments. AMP, antimicrobial peptide.

Results of the checkerboard titrations for combinations of ChBac3.4, PG-1, and lysozyme with gelatin-stabilized silver nanoparticles against drug-susceptible bacteria are represented as isobolograms at Figure 3. Minimal FICIs are shown in the upper right corners of the corresponding plots. They are typed in green for synergy, in blue for additive effect, and in violet for independent action. Additional red numbers on the plots are FICIs calculated for combinations corresponding to red dots. MIC values for antimicrobial action of gelatin-stabilized silver nanoparticles alone are given under the isobolograms as medians of 3–4 independent broth microdilution tests.

According to the obtained data, combined effect of PG-1 and lysozyme with silver nanoparticles proves to be synergistic in almost all cases, except that against *A. baumannii* for lysozyme. ChBac3.4 in combination with nanoparticles mainly shows additivity.

Although the combined action with AMPs for the gelatin-stabilized nanoparticles was not further examined against multidrug-resistant isolates, we carried out such experiments using the combinations of PG-1 or ChBac3.4 with poviargolum (Table 5), which is a preparation of polyvinylpyrrolidone-stabilized highly dispersed silver used as broad-spectrum bactericidal agent (Patent of the Russian Federation No 2088234, owned by the Institute of Macromolecular Compounds RAS). Synergy was found with PG-1 against Gram-negative clinical isolates *P. aeruginosa* MDR 522/17, *K. pneumoniae* ESBL 344/17 and *A. baumannii* 7226/16, and, interestingly, with ChBac3.4 as well: against Gram-negative strains *E. coli* ESBL 521/17, *K. pneumoniae* ESBL 344/17, and Gram-positive bacterium *S. aureus* 1399/17.

**Table 5:** Antimicrobial activity of colloidal silver preparation “Poviargolum” alone and in combinations with PG-1 or ChBac3.4 against drug-resistant clinical isolates.

**Bacteria**	**Poviargolum MIC^a^ (μg/ml)**	**Poviargolum and PG-1 FICI^b^**	**Poviargolum and ChBac3.4 FICI^b^**
*E. coli* ESBL 521/17	78	0.56	**0.375**
*A. baumannii* 7226/16	78	**0.5**	0.75
*P. aeruginosa* MDR 522/17	78	**0.5**	0.625
*K. pneumoniae* ESBL 344/17	78	**0.5**	**0.5**
*S. aureus* 1399/17	156	0.62	**0.5**

a *Minimal inhibitory concentrations (MIC) values are medians of 4 independent experiments made in triplicates*.

b *Fractional Inhibitory Concentration Indices (FICI) values are medians of 3–4 independent experiments; FICI > 2 indicates antagonism, 1 < FICI ≤ 2 shows independent action, 0.5 < FICI ≤ 1 corresponds to additivity, FICI ≤ 0.5 denotes synergy; synergy cases are set off in bold type*.

#### Analysis of the Bacterial Membrane Permeabilization

**Figure 4:**
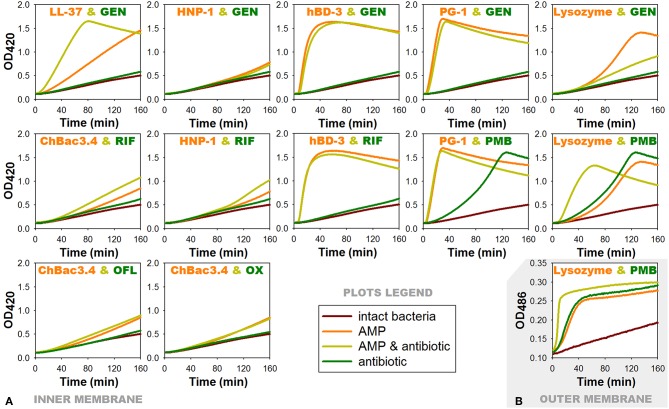
Effects of combinations of AMPs with antibiotics showing antibacterial synergy on membrane permeability of *E. coli* ML-35p. **(A)** Permeability of the inner membrane for a chromogenic marker o-nitrophenyl-β-D-galactoside (ONPG). ¼ of minimal inhibitory concentrations (MICs) of antimicrobial peptides (AMPs) and antibiotics were used when alone, and ¼ MIC of AMP + ¼ MIC of antibiotic when in combination. Curves illustrate ONPG degradation by the cytoplasmic β-galactosidase of bacteria, when the membranes are damaged by test substances. Curves slope and the time of their rising to the plateau correspond to the extent of this damage. **(B)** Permeability of the outer membrane for a chromogenic marker nitrocefin under the effect of combination of lysozyme with polymyxin B. Outer membrane permeability was examined similarly to the inner membrane except for the chromogenic marker being used was nitrocefin in concentration of 20 μM and the OD measurement was performed at 486 nm. Nitrocefin is a substrate for the periplasmic β-lactamase of the bacteria. RIF, rifampicin; PMB, polymyxin B; GEN, gentamicin; OFL, ofloxacin; OX, oxacillin.

Plots at Figure 4A show obtained kinetic curves, illustrating the changes in the permeability of the cytoplasmic bacterial membrane caused by AMP/antibiotic combinations, which most often demonstrated synergy of antibacterial action, in comparison with the effect of individual compounds. We examined combined effect of ChBac3.4 with rifampicin, ofloxacin, or oxacillin; of PG-1 or LL-37 with gentamicin; of HNP-1 or hBD-3 with gentamicin or rifampicin; and of lysozyme with polymyxin B. However, it should be mentioned, that not all of these combinations demonstrated synergy specifically against *E. coli* ML-35p. In addition, a combination of two membranolytic agents PG-1 and polymyxin B, whose interaction was predictably additive, and a combination of lysozyme and gentamicin, whose effect was close to synergistic, were also considered.

For AMP/antibiotic pairs, where the peptide component demonstrates rapid membranolytic effect, such as hBD-3/gentamicin, hBD-3/rifampicin and PG-1/gentamicin, curves of combined action almost replicate those illustrating the action of the corresponding peptide alone. In case of the additive combination of PG-1 with polymyxin B, where both components are distinctly membranoactive, total effect is predictably equal to the individual action of PG-1, as it arises more quickly.

The effect of lysozyme and polymyxin B, when they are applied in combination, is noticeably intensified: its development rate doubles. Interestingly, the same phenomenon could be observed for the outer membrane of the tested bacteria (Figure 4B) using another chromogenic marker nitrocefin, which is a substrate of *E. coli* ML-35p periplasmic β-lactamase. The latter may indicate that the interaction between lysozyme and polymyxin B is not limited to the facilitation of lysozyme access to the peptidoglycan layer. It is possible that lysozyme in its turn contributes directly to the process of polymyxin interaction with the outer membrane of bacteria, and later with the inner membrane as well.

ChBac3.4 and HNP-1 show no substantial effect on the membrane permeability in concentrations equal to ¼ MIC, which is expected, as dimeric pores suggested for HNP-1 do not cause significant membrane disruption (Lehrer and Lu, 2012), and ChBac3.4 in low concentrations is believed to act via non-membranolitic mechanism (Shamova et al., 2009). However, slight acceleration in the development of membrane-damaging effect of these peptides, when in combination with rifampicin, can be noted.

Significantly more prominent effect of this nature occurs for the combination of LL-37 with gentamicin. AMP alone is causing quite slow enhance of the membrane permeability, but in presence of gentamicin the process of membrane-damaging is developing rather quickly. In the tested concentration gentamycin alone causes no substantial increase in the permeability of the membrane. It means that a non-specific membrane damaging, which is possible at the late stages of aminoglycosides action on bacteria, when the protein synthesis is affected and incorrectly synthesized proteins are embedded into the membrane disturbing its stability (Taber et al., 1987), is out of the picture. Perhaps, aminoglycoside molecules can contribute to the lipid clusterization process and overall membrane disturbance, thereby accelerating AMP integration into the bilayer and/or their aggregation.

For another combination with gentamicin, which includes α-defensin HNP-1, no changes are found comparing the action of the peptide alone or with the antibiotic. As it was mentioned, HNP-1 does not show prominent effect on the permeability of bacterial membrane in concentrations of ¼ MIC. Though, synergy between HNP-1 and gentamicin is observed not against *E. coli* ML-35p, but against Gram-positive bacteria *M. luteus* CIP A270. Probably, in this case a certain role in the realization of the synergistic effect can be attributed to the ability of defensins (in particular, HNP-1) to bind lipid II and block the biosynthesis of the cell wall, described not so long ago (de Leeuw et al., 2010).

In case of lysozyme, the addition of gentamicin in the mixture unexpectedly results in slower increase of permeability in comparison to the action of lysozyme alone. The effect of the combination against *E. coli* ML-35p is close to synergy, though the bacterium is highly resistant to lysozyme, as it is Gram-negative. It could be speculated, that after lysozyme penetrates through the outer membrane of *E. coli* ML-35p, it induces the cleavage of the cell wall, and the access of the gentamicin molecules to its targets is probably facilitated, similar to the mechanism described for aminoglycosides and β-lactam antibiotics. However, to explain observed changes in the dynamic of lysozyme-induced membrane permeability increase, further research is needed. Probably, the non-enzymatic mechanism, reported for lyzozyme (Laible and Germaine, 1985; Ibrahim et al., 2001) is involved.

### Effects on Eukaryotic Cells

#### Combined Hemolytic Activity

Synergistic antimicrobial combinations of PG-1, ChBac3.4, lysozyme, and LL-37 with antibiotics (from Table 2) were tested for hemolytic activity. MECs for individual substances are summarized in Table 6. Peptides PG-1 and LL-37 start to show hemolytic properties in quite low concentrations of <10 μM in *in vitro* conditions in absence of blood serum; amongst the antibiotics only oxacillin and rifampicin demonstrate slight hemolityc action. Results for combinations in Table 7 illustrate by “+” the presence or by “–” the absence of any statistically significant hemolytic effect of examined mixtures of AMPs with antibiotics in a series of three independent experiments. Based on these data the corresponding FECI value for each combination is assessed. Despite hemolytic properties possessed by some of the tested compounds, no cases of synergy or even additivity regarding combined hemolytic action are detected.

**Table 6:** Cytotoxic action of individual fractions of AMPs and antibiotics toward normal or tumor mammalian cells and their hemolytic activity toward human erythrocytes.

**Sample**	**MEC^a^ (μM) of cytotoxic action toward**					**MEC^a^ (μM) of hemolysis of human erythrocytes**
	**Normal Cells**			**Tumor Cells**		
	**Human PBMC**	**Human neutrophils**	**Murine peritoneal macrophages**	**K562**	**Murine EAC**	
PG-1	5.0	2.5	2.5	1.25	1.25	2.0
LL-37	12	10	>20 [40]	5.0	10	5.0
Lysozyme	>50 [100]	>50 [100]	>50 [100]	>50 [100]	50	>50 [100]
ChBac3.4	20	>20 [40]	>20 [40]	20	5.0	>50 [100]
Gentamicin	>50 [100]	50	>50 [100]	50	>50 [100]	>50 [100]
Rifampicin	>20 [40]	20	>20 [40]	>20 [40]	>20 [40]	20
Oxacillin	>50 [100]	>50 [100]	>50 [100]	>50 [100]	>50 [100]	25
Ofloxacin	>50 [100]	>50 [100]	>50 [100]	>50 [100]	>50 [100]	>50 [100]
Polymyxin B	50	>50 [100]	50	25	50	>50 [100]

*PBMC, peripheral blood mononuclear cells; EAC, Ehrlich ascites carcinoma*.

a *Minimal effective concentrations (MEC) values are medians of 3–4 independent experiments made in triplicates. MECs are minimal concentrations where the statistically significant difference from the untreated (intact) cells is found with Mann-Whitney U-test (p < 0.01; n_1_ = 3, n_2_ = 6–8 for cytotoxic action; p < 0.05, n_1_, n_2_ = 3 for hemolysis). If actual MEC value was out of the tested concentrations range, it was assessed as twice the maximal tested concentration; the corresponding value is given in square brackets*.

**Table 7:** Hemolytic action of combinations of AMP and conventional antibiotics toward human erythrocytes.

**A and B combination**	**Hemolytic activity**		**FECI^a^**
	**(½ MEC A + ½ MEC B)**	**(¼ MEC A + ¼ MEC B)**	
LL-37 and GEN	+ – – [–]	– – – [–]	>1.0
PG-1 and GEN	– – – [–]	– – – [–]	>1.0
Lysozyme and GEN	– – – [–]	– – – [–]	>1.0
Lysozyme and PMB	– – – [–]	– – – [–]	>1.0
ChBac3.4 and OX	– – – [–]	– – – [–]	>1.0
ChBac3.4 and OFL	– – – [–]	– – – [–]	>1.0
ChBac3.4 and RIF	– – – [–]	– – – [–]	>1.0

*GEN, gentamicin; PMB, polymyxin B; OX, oxacillin; OFL, ofloxacin; RIF, rifampicin. + | – presence or absence of the statistically significant difference from intact cells (Mann-Whitney U-test p < 0.05, n_1_, n_2_ = 3) in each of the three independent experiments; resulting assessment of the combination effect is given in square brackets*.

a *Assessment of the minimal Fractional Effective Concentration Index (FECI) based on the results for (½ MEC A + ½ MEC B) and (¼ MEC A + ¼ MEC B) combinations of substances A and B; FECI > 1.0 shows independent action or antagonism, 0.5 < FECI ≤ 1 indicates additivity, FECI ≤ 0.5 denotes synergy. MEC, minimal effective concentration*.

#### Combined Cytotoxic Action

To determine whether the cytotoxicity of combinations of AMPs and antibiotics against various normal and tumor eukaryotic cells is higher than that of individual substances, we used MTT-test. The effect was observed toward human PBMC and neutrophils of healthy donors, murine peritoneal macrophages of healthy mice, human erythromyeloid leukemia cell line K562 and murine EAC cells. Examined combinations were the same as for the hemolytic assay. Additionally, the combination of PG-1 and polymyxin B, where both compounds had explicit membranolytic action, was tested.

MECs of individual substances were determined before examining combined action (Table 6). Though AMPs are more toxic toward tumor cells, which correspond to the literature (Al-Benna et al., 2011; Gaspar et al., 2013), PG-1 also starts to damage normal cells in quite low concentrations. To the lesser extent it is true for LL-37 against human PBMC and neutrophils, and ChBac3.4 against human PBMC. Antibiotics are generally non-toxic toward both tumor and normal eukaryotic cells in concentrations below 25–50 μM.

Data on the toxicity of AMP/antibiotic combinations in three independent experiments and corresponding FECIs values are summarized in Table 8 in the same manner as for the hemolysis. Despite the promising results of the hemolytic test, we identified synergy of cytotoxic action against tumor and/or normal cells in a number of cases. Thus, LL-37/gentamicin combination provides synergistic effect toward both types of tested tumor cells (human K562, murine EAC) and normal PBMC; toward human neutrophils and murine peritoneal macrophages it shows at least additive interaction. Combination of lysozyme with polymyxin B also demonstrates synergy against K562, EAC and PBMC cells, and additivity toward peritoneal macrophages of mice. PG-1 and gentamicin applied together exert synergistic cytotoxic action on human neutrophils; however, in other cases the effect is independent. Combination of PG-1 and polymyxin B shows synergy against K562 cells, and PMBC. The combined effect of lysozyme and gentamicin on eukaryotic cells is additive in the majority of the cases. For combinations of ChBac3.4 and antibiotics, combined action is mainly independent.

**Table 8:** Cytotoxic action of combinations of AMP and conventional antibiotics toward normal or tumor mammalian cells.

**A and B**	**Toxicyty of (**½ **MEC A** **+** ½ **MEC B) and (**¼ **MEC A** **+** ¼ **MEC B) combinations and corresponding FECIs^^a^^**														
	**Normal cells**									**Tumor cells**					
	**Human PBMC**			**Human neutrophils**			**Murine peritoneal macrophages**			**K562**			**Murine EAC**		
	**½ A and ½ B**	**¼ A and ¼ B**	**FECI**	**½ A and ½ B**	**¼ A and ¼ B**	**FECI**	**½ A and ½ B**	**¼ A and ¼ B**	**FECI**	**½ A and ½ B**	**¼ A and ¼ B**	**FECI**	**½ A and ½ B**	**¼ A and ¼ B**	**FECI**
LL-37 and GEN	+ + + [+]	+ + + [+]	**0.5**	+ + + [+]	+ – – [–]	1.0	+ + + [+]	– – – [–]	1.0	+ + + [+]	+ + + [+]	**0.5**	+ + + [+]	+ + + [+]	**0.5**
PG-1 and GEN	– – – [–]	– – – [–]	>1.0	+ + + [+]	+ + – [+]	**0.5**	– – – [–]	– – – [–]	>1.0	– – – [–]	– – – [–]	>1.0	– – – [–]	– – – [–]	>1.0
PG-1 and PMB	+ + + [+]	+ + + [+]	**0.5**	+ – – [–]	– – – [–]	>1.0	+ + + [+]	– – – [–]	1.0	+ + + [+]	+ + – [+]	**0.5**	+ + + [+]	+ – – [–]	1.0
LYZ and GEN	+ – – [–]	– – – [–]	>1.0	+ + + [+]	+ – – [–]	1.0	– – – [–]	– – – [–]	>1.0	+ + + [+]	– – – [–]	1.0	+ + + [+]	+ – – [–]	1.0
LYZ and PMB	+ + + [+]	+ + + [+]	**0.5**	+ – – [–]	– – – [–]	>1.0	+ + + [+]	– – – [–]	1.0	+ + + [+]	+ + + [+]	**0.5**	+ + + [+]	+ + + [+]	**0.5**
ChBac3.4 and OX	+ + + [+]	+ – – [–]	1.0	+ – – [–]	– – – [–]	>1.0	– – – [–]	– – – [–]	>1.0	– – – [–]	– – – [–]	>1.0	– – – [–]	– – – [–]	>1.0
ChBac3.4 and OFL	– – – [–]	– – – [–]	>1.0	+ – – [–]	– – – [–]	>1.0	– – – [–]	– – – [–]	>1.0	– – – [–]	– – – [–]	>1.0	– – – [–]	+ – – [–]	>1.0
ChBac3.4 and RIF	– – – [–]	– – – [–]	>1.0	– – – [–]	– – – [–]	>1.0	– – [–]	– – – [–]	>1.0	– – – [–]	– – – [–]	>1.0	– – – [–]	– – – [–]	>1.0

*GEN, gentamicin; PMB, polymyxin B; OX, oxacillin; OFL, ofloxacin; RIF, rifampicin; LYZ, lysozyme*.

*+ | – presence or absence of the statistically significant difference from intact cells (Mann-Whitney U-test p < 0.01; n_1_ = 3, n_2_ = 6–8) in each of the three independent experiments; resulting assessment of the combination effect is given in square brackets*.

a *Assessment of the minimal Fractional Effective Concentration Index (FECI) based on the results for (½ MEC A + ½ MEC B) and (¼ MEC A + ¼ MEC B) combinations of substances A and B; FECI > 1.0 shows independent action or antagonism, 0.5 < FECI ≤ 1 indicates additivity, FECI ≤ 0.5 denotes synergy; synergy cases are set off in bold type. MEC, minimal effective concentration*.

We noticed that in the identified synergistic combinations at least one component demonstrates a pronounced membranolythic action on bacterial membranes (PG-1, LL-37, polymyxin B). This fact corresponds with generally accepted conception that such mechanism contributes to non-specific toxicity toward eukaryotic cells as well.

The obtained data indicate that combined use of AMP and antimicrobial antibiotics in some cases can result in simultaneous enhancement of antibacterial action and of cytotoxic effects on eukaryotic cells. Therefore, to strengthen only the first kind of activity, the selection of effective combinations should be approached with caution. However, it should be noted that in the majority of cases the concentrations of conventional antibiotics used to examine the combined action on eukaryotic cells significantly exceeded those used to study the combined effect on bacteria. Resulting difference in the molar ratios of the components between combinations tested on eukaryotic and bacterial cells probably makes the current assessment a bit more pessimistic than it should have been, if component ratios were preserved in accordance to antimicrobial assays.

At the same time, combined cytotoxic action of PG-1 with anticancer agent doxorubicin was determined to be synergistic against both doxorubicin-sensitive and doxorubicin-resistant K562 tumor cells with minimal FECIs being 0.25 and 0.38, respectively (median values based on three independent experiments). However, it must be noted, that this phenomenon was tested regarding minimal toxicity level, and at the 50%-effect level used to define IC_50_ in standard antitumor activity tests the type of interaction may differ. It is also of interest, that the enhancement in MEC values comparing susceptible and resistant cell lines is significantly higher for doxorubicin than for PG-1 (100 times, from 1 to 100 μg/ml, for doxorubicin against 4 times, from 1.25 to 5.0 μM, for PG-1). However, it may simply be due to the absence of cross-resistance.

## Discussion

AMPs of animals' host defense are widely acknowledged to effectively suppress the growth of microorganisms resistant to the clinically used drugs (Hancock and Lehrer, 1998; Deslouches et al., 2013; Kang et al., 2014). The synergy of various AMPs with different antibiotic compounds including other AMPs or proteins of neutrophil granules as well as clinically used antibiotics has been reported in numerous papers (Giacometti et al., 2000a,b,c; Yan and Hancock, 2001; Cassone and Otvos, 2010; Yu et al., 2016). Current research was aimed to study the combined effects of AMPs with other antibiotic agents considering their direct antibacterial and cytotoxic activity in order to reveal certain patterns in such interactions that can provide additional information on the mechanisms standing behind the observed synergistic action, and to verify the effectiveness of such combinations against multidrug-resistant bacterial strains.

A number of different generalized models explaining synergy are in place. Some are specific to the mechanisms of action of both components. They applies to the cases when the ways in which interacting compounds perform their individual action converge at some specific site; for example, if two drugs are inhibiting alternative pathways producing the same essential metabolite (Jia et al., 2009; Yeh et al., 2009), or if they share the same target, but bind non-competitively, providing synergy on molecular level (Breitinger, 2012). In other scenarios synergistic action is contributed mostly to the mechanism of action of one of the components in the pair. In such cases this component provides to the other a better ability to exert its effect (Zimmerman et al., 2007). It can be either due to facilitating the access of the second component to its targets or due to inhibiting some mechanism, for example, biodegradation, that prevent the second component from taking its action (Zimmerman et al., 2007; Cokol et al., 2011). In case of this bioavailability model, the first compound which is a “provider” of said bioavailability can demonstrate synergistic interactions with a large number of substances having distinctly different mechanisms of action (Chou, 2006). On the other hand, some researchers point out a possible danger of increasing bioavailability not only for the desired drug, but also for other substances which were not taken into consideration (Cokol et al., 2011).

Synergy observed so far in AMP/antibiotic pairs or for AMPs with antimicrobial proteins co-located with them *in vivo* is generally attributed to AMPs brand ability to enhance the permeability of bacterial membranes and by extent the access of other compounds into the periplasm and cytoplasm of bacterial cells (Cassone and Otvos, 2010; Yenugu and Narmadha, 2010; Singh et al., 2014; Feng et al., 2015; Gupta et al., 2015; Khara et al., 2015; Soren et al., 2015). However, it do not eliminate the possibility that synergistic interactions of AMPs with other antimicrobials are not limited to just that.

Most of the cases of synergistic interaction we found in present study were between AMPs having a pronounced effect on the permeability of bacterial membranes (PG-1, β-defensins, LL-37) and antibiotics affecting the biosynthesis of nucleic acids and proteins, which, in order to perform their microbicidal action, must penetrate inside the cell. This fact supports the existing bioavailability model proposed for such synergy, suggesting the crucial role of AMP-provided membranolytic action in it.

Most frequently synergy was observed in combinations of AMPs with aminoglycoside gentamycin affecting protein synthesis at ribosomes, whereas effects of combined action with antibiotics influencing nucleic acid synthesis (rifampicin and ofloxacin) were a bit less frequent (more so for ofloxacin). The rate of development of the damaging effect can play a certain role here: violation of the synthesis of nucleic acids affects cell viability in a more distant perspective and needs more time to make a significant contribution compared to the rapid damage caused by the peptide, and compared to the peptide synthesis blockage on ribosome as well. Another factor may be the presence of rigid structures of fused aromatic rings in ofloxacin and rifampicin molecules which can limit to some extent their ability to penetrate through the pores formed by AMPs.

Synergy with gentamycin was reproduced with another aminoglycoside amikacin. According to existing knowledge, after the electrostatic binding of aminoglycoside molecules carrying large positive charge to the negatively charged structures distributed on the surface of bacteria, the energy-dependent uptake of antibiotic into the cell takes place, although no specific carrier proteins were reported (Taber et al., 1987). Frequently reported cases of synergy between aminoglycosides and β-lactam antibiotics (Rahal, 2006; Leibovici and Paul, 2007) are believed to occur due to the damaging of the cell wall by β-lactams, facilitating the access of aminoglycosides to their targets (Davis, 1982). Based on that, the antimicrobial synergy of aminoglycosides with AMPs can also be attributed to the main proposed model: membrane-damaging performed by peptide allows easier access into the cell for the antibiotic. The fact that the synergistic action of gentamicin and membranolytic AMP PG-1 is more pronounced against Gram-negative bacteria (Table 2; Figure 1), where besides the cytoplasmic membrane, an additional external one also exists, supports this theory.

The scenario of bioavailability increase is also applicable to the combination of lysozyme and polymyxin B found to exert synergy against Gram-negative bacteria. Our data correspond with the known fact, that the pretreatment with non-bactericidal doses of polymyxin B allows lysozyme to lyse bacterial wall of Gram-negative bacteria in concentrations which are otherwise ineffective (Warren et al., 1957), and supply further evidence for this synergy in formal terms of Loewe additivity model. However, in this case it is the antibiotic that presumably damages the outer membrane to provide lysozyme with the access to the peptidoglican layer. This hypothesis is supported by the study of combined action of lysozyme and polymyxin B immobilized on agarose beads which prevented the latter from interacting with other bacterial structures except outer membrane (Rosenthal and Storm, 1977). Though, there are facts demonstrating that the bioavailability model here works the other way around as well. The report by Galizzi et al. (1975) indicates that the presence of low doses of lysozyme, which do not affect microbial growth, renders Gram-positive bacteria *Bacillus subtilis*, both polymyxin-resistant and sensitive strains, susceptible to 10 times lower concentrations of polymyxin B. And the dynamic of permeabilization of the cytoplasmic membrane of *E. coli* ML-35p by polymyxin B observed in current study shows significant acceleration in presence of lysozyme.

The effect of combinations on the permeability of bacterial membranes in comparison with the effects of individual substances in the same concentrations was investigated not only for lysozyme and polymyxin B, but also for other AMP/antibiotic pairs showing synergy, as the damage to bacterial membranes is simultaneously the main mechanism of antibacterial action of AMPs and the main estimated cause of their synergistic interaction with other antimicrobial compounds.

From mechanistic considerations if the synergy-providing interaction of AMP and antibiotic is limited to the easier access of the latter into the inner space of bacterial cells using AMP-formed pores in their membranes, the effect of AMP on the membrane permeability should not be affected by the presence or absence of antibiotic, at least in the beginning when antibiotic impact on bacterial biosynthesis is yet to manifest. This assumption proved to be right for AMP/antibiotic pairs including PG-1 and hBD-3 which caused swift increase of membrane permeability.

On the other hand, observed synergy cases were not limited to combinations with AMPs demonstrating rapid membranolytic action in tested concentrations. Thus, ChBac3.4 and HNP-1 in tested concentrations caused only slight effect on cytoplasmic membrane permeability. In case of synergy between HNP-1 and gentamicin against Gram-positive bacteria, HNP-1 described ability to bind lipid II, thereby disturbing bacterial cell wall synthesis, can contribute to the interaction observed, as in mentioned synergy of aminoglycosides with β-lactams (Rahal, 2006; Leibovici and Paul, 2007) the latter also affect said synthesis. Some supplementary mechanisms could be involved for ChBac3.4 as well, as its action is believed to include intracellular targets in addition to membrane disruption, especially in low concentrations.

Another interesting observation is a prominent acceleration of the membrane damaging process compared to the action of AMP alone found for the combination of LL-37 with gentamicin. On a lesser scale such effect was also observed for combinations of ChBac3.4 and HNP-1 with rifampicin. It suggests that in some cases the interaction between AMP and antibiotic may be more complex than the penetration of the antibiotic through the pores formed by peptide, and can include some direct or indirect effect on the incorporation or orientation of AMP molecules in the bacterial membrane mediated by antibiotic.

Further examination of antibiotics combinations with PG-1 and ChBac3.4 against clinically isolated multidrug-resistant strains of *E.coli, A. baumanii, P. aeruginosa, K. pneumoniae*, and *S. aureus* confirmed the described above dependencies in synergistic interaction. Most numerous cases of synergy were found for both peptides with amikacin used instead of gentamicin in these tests, and with erythromycin. This two antibiotics both affect protein synthesis by binding to bacterial ribosomes. Other studies also report synergy of AMPs with antibiotics targeting this process, such as chloramphenicol (Zhang et al., 1999; Yenugu and Narmadha, 2010; Sanchez-Gomez et al., 2011; Rajasekaran et al., 2017); tetracyclines (Giacometti et al., 2000c; Yenugu and Narmadha, 2010; Sanchez-Gomez et al., 2011); aminoglycosides gentamicin (Yenugu and Narmadha, 2010; Wu et al., 2017), tobramycin (Payne et al., 2017; Pollini et al., 2017), kanamycin (Anantharaman et al., 2010; Yenugu and Narmadha, 2010), streptomycin (Yenugu and Narmadha, 2010); macrolides azithromycin (Wu et al., 2017), erythromycin (Vaara and Porro, 1996; Ulvatne et al., 2001; Moerman et al., 2002; Sanchez-Gomez et al., 2011; Gopal et al., 2014; Jindal et al., 2017), clarithromycin (Giacometti et al., 1999, 2000a,b,c). Synergistic interactions with rifampicin affecting transcription and hence RNA synthesis are also frequently described (Vaara and Porro, 1996; Giacometti et al., 2000a; Ulvatne et al., 2001; Cirioni et al., 2008; Anantharaman et al., 2010; Yenugu and Narmadha, 2010; Khara et al., 2014, 2015; Gupta et al., 2015; Soren et al., 2015; Pollini et al., 2017).

Synergy with ofloxacin disturbing DNA-replication process was also shown against drug-resistant isolates, thought for PG-1 only against ofloxacin-sucseptible *K. pneumoniae*. Synergistic interactions with different quinolone antibiotics are indicated in various publications: with naladixic acid (Scott et al., 1999; Zhang et al., 1999; Sanchez-Gomez et al., 2011), with ciprofloxacin (Scott et al., 1999; Giacometti et al., 2000a; Yenugu and Narmadha, 2010; Gopal et al., 2014; Singh et al., 2014; Bessa et al., 2018), with levofloxacin (Feng et al., 2015), and with norfloxacin (Niu et al., 2013); as well as synergy with novobiocin which also inhibit DNA-gyrase activity (Vaara and Porro, 1996).

Although the indications are quite vague, synergy between ChBac3.4 and oxacillin seems to manifest specifically against *S. aureus* strains resistant to this antibiotic. Further study is required to clarify if there are some unique features underlying AMP/antibiotic interaction in this case. Noteworthy, not only ChBac3.4, but also PG-1 was found to be synergistic with meropenem, another β-lactam antibiotic, against resistant *A. baumanii* and moderately resistant *S.aureus*; however, against meropenem-resistant *P. aeruginosa* this effect wasn't shown. Nevertheless, the obtained data look promising in the light of recent challenges posed by carbapenem-resistant bacteria. For both oxacillin and meropenem, synergy was found against bacteria which had enhanced MIC levels to these antibiotics, and was not found against those more sensitive; thereby it may be suspected, that some resistance mechanism can be affected by AMPs, thought further investigation is needed. Cases of AMPs synergy with β-lactam antibiotics against multidrug-resistant bacteria (including those resistant to this particular β-lactam) are also demonstrated in numerous works for ampicillin (Yenugu and Narmadha, 2010; Sanchez-Gomez et al., 2011), amoxicillin (Giacometti et al., 2000c; Moerman et al., 2002; Sanchez-Gomez et al., 2011; Wu et al., 2017), carbenicillin (Scott et al., 1999; Yenugu and Narmadha, 2010), ticarcillin (Sanchez-Gomez et al., 2011), aztreonam (Pollini et al., 2017), piperacillin (Giacometti et al., 2000b,c), cephalosporins ceftazidime (Giacometti et al., 2000b,c; Soren et al., 2015; Bessa et al., 2018), cefuroxime (Moerman et al., 2002), ceftriaxone (Giacometti et al., 2000c; Singh et al., 2014; Soren et al., 2015; Jindal et al., 2017), cefepime (Feng et al., 2015), cefotaxime (Gopal et al., 2014; Singh et al., 2014); carbapenems meropenem (Giacometti et al., 2000b,c; Sanchez-Gomez et al., 2011; Pollini et al., 2017), and imipenem (Sanchez-Gomez et al., 2011; Feng et al., 2015). In addition, AMPs are reported to synergize with vancomycin (Giacometti et al., 2000a; Shin et al., 2002; Feng et al., 2015; Wu et al., 2017) which also disturbs cell wall synthesis. He et al. (2015) indicate that wide synergy between magainin II and β-lactam antibiotics, also observed by Giacometti et al. (2000c), seems to be unusual, and may be provided by some unique mechanism. Giacometti et al. (2000a,b) mention the hypothesis that AMPs may trigger the activity of bacterial murein hydrolases, hence contributing to the peptidoglycan degradation in cooperation with β-lactams. Regarding possible resistance mechanisms attenuation, Sanchez-Gomez et al. (2011) suggest that some AMPs can counteract efflux pump overexpression. Soren et al. (2015) also refer to efflux pump systems as possible targets of AMPs action in Gram-negative bacteria.

Interestingly, in as much as 72.8 % (16 of 22) of all synergy cases observed in clinical isolates in the current study, bacteria indeed showed moderate or high level of resistance toward the antibiotic taking part in the synergistic pair. Thus, combined use of AMPs and antibiotics could be a valuable tool to decrease MIC levels of antibiotics in resistant strains, even if only 4–16 times. The ability of AMPs to prevent biofilm formation or affect those already formed (Algburi et al., 2017; Chung and Khanum, 2017) can also contribute to overcoming bacterial resistance to antibiotics. This kind of potentiation of antimicrobial activity was suggested in several reports (Gopal et al., 2014; de la Fuente-Núñez et al., 2015; Bessa et al., 2018) further substantiating possible benefits of combination antimicrobial therapy including AMPs. However, our study and previous works (Pollini et al., 2017) indicate that observed synergy is not universal for combating bacterial strains and species with different resistance profiles. Hence, such combination therapy will not allow avoiding susceptibility testing procedures even if applied in healthcare practice.

It is also of note, that in synergy cases with conventional antibiotics we observed *in vitro* for human AMPs their concentrations were around 150 nM for LL-37, 12.5–125 nM for HNP-1, 0.2–1.6 μM for hBD-2, and 0.08–3.75 μM for hBD-3. Some of these values fall within the range of concentrations of said peptides in different body fluids of healthy individuals reported in literature. It is mostly true for LL-37, which was detected in concentration of 0.15–6.1 μM in saliva, of 0.16–1.9 μM in bronchoalveolar lavage (BAL), and of 0.2–0.5 μM in plasma (Byfield et al., 2011), and to the lesser extent for α-defencin, which was found in plasma in concentration of around 80–95 nM [13.5 ± 1.2 ng/50 μL according to Mattar et al. (2016) and 323.3 ± 173.1 ng/ml according to Mukae et al. (2002)] and in BAL in concentration of 3.7 nM (12.9 ± 15) ng/ml according to Mukae et al. (2002). However, for β-defensins reported concentrations were lower, in saliva their detected levels were 2.2 (0.3–4.8) nM [9.5 (1.2–21) μg/L] for hBD-2 and 63 (10–182) nM [326 (50–931) μg/L] for hBD-3 (Ghosh et al., 2007), hBD-2 concentration in serum was reported to be only 8.3 ± 3.9 pM (36.1 ± 17.0 pg/ml) (Arimura et al., 2004), and hBD-3 was not detected in BAL (Ghosh et al., 2007). Though, local concentrations of AMPs in the site of inflammation are supposed to become significantly higher, than these mean values.

We also found synergy of antibacterial action in combinations of the membranolytic AMP PG-1 and of the antimicrobial protein lysozyme with gelatin-stabilized silver nanoparticles, when tested on laboratory strains. This effect was observed in the majority of cases, against both Gram-positive and Gram-negative bacteria, and was quite pronounced providing 8–16 times decrease of effective concentrations. Synergy was previously described for silver nanoparticles with polymyxin B (Ruden et al., 2009), which acts rather similarly to the membranolitic AMPs. However, for ChBac3.4 interaction was additive as in some reported cases for other AMPs (Ruden et al., 2009; Mohanty et al., 2013). The impact of silver nanopaticles used in our study on bacterial membranes permeability was rather slow for the outer and absent for the inner one (Supplementary Figure S1A), but their effect on metabolic activity of bacteria was swift (Supplementary Figure S1B), suggesting that they exert their action without penetrating through the cytoplasmic membrane. However, based on the observed synergy cases, we assume that membranolytic activity of AMPs plays a certain role in the realization of synergistic interaction. We could also hypothesize, that wide-scale oxidative effects provided by silver compounds can contribute to this outcome, as it has been reported that oxidation of membrane lipids potentiated bactericidal activity of membranoactive AMPs (Libardo et al., 2017).

The preparition of colloid silver stabilized with polyvinylpyrrolidone, currently used as a bactericidal agent in Russian Federation, also exerted synergistic effect of antimicrobial action in combination with AMPs against multidrug-resistant clinical isolates, including Gram-negative carbapenem-resistant strains of *A. baumanii* and *P. aeruginosa*, when with PG-1, and mildly meropenem-resistant Gram-positive strain *S. aureus* 1399/17, when with ChBac3.4; though only 4 times dose reduction was achieved in these cases.

Overall, the phenomenon of mutual potentiation of antibacterial activity for combinations of silver nanoparticles or colloids with antimicrobial polypeptides manifests rather abundantly. As well as AMPs, silver nanoparticles are believed to provide wide-scale damage for bacterial cells (Dakal et al., 2016). Bacterial resistance to silver preparitions is rarely induced (Yang et al., 2018). Both AMPs (Algburi et al., 2017; Chung and Khanum, 2017), and nanoparticles (Blanchette and Wenke, 2018) are reported to prevent biofilm formation. Considering all these facts, the possibility of combined use of these two classes of compounds against multidrug-resistant pathogens looks very promising. As the penetration within bacterial cytoplasm do not appear to be necessarily required for the successful action of named substances, the idea of developing AMP-capped silver nanoparticles also has a prospect of practical use, as it was already suggested for polymyxin B stabilized ones (Lambadi et al., 2015).

Though hints on the additional mechanisms behind the synergy of AMPs with other antimicrobial compounds were found, their effect on bacterial membranes permeability seems to be the main (or at least very important) cause of said interaction. The wide-scale effect provided by AMPs on membrane bilayers has its downside of comparatively low selectivity and is believed to be the cause of some cytotoxic effects toward eukaryotic cells, for instance, hemolytic activity, observed in many AMPs (Matsuzaki, 2009; Takahashi et al., 2010). Hence, we considered possible amplification of toxicity in combinations, as several cases where antibiotic presence enhanced the rate of membrane damaging caused by AMP in bacteria had been revealed in previous experiments. The results of hemolytic test for AMP/antibiotic combinations showing antimicrobial synergy were promising: no evidence of synergy or even additivity of hemolytic action was detected, supporting the notion that combined use may indeed reduce toxicity and increase selectivity. The same is reported for AMPs and silver nanoparticles (Ruden et al., 2009). However, when cytotoxicity was tested against different eukaryotic cells using MTT-test, combinations including membranoactive components were found synergistic in a number of cases. Though the proportion of components in tested combinations was significantly different than that in the antimicrobial assays, as the range between toxic and antimicrobial concentrations is wider for antibiotics than for AMPs, observed synergy indicates that this factor should be closely considered when choosing the optimal antimicrobial compositions containing AMPs. On the other hand, ability of AMPs to synergistically enhance cytotoxic effects in combinations with other compounds can be of use regarding possible antitumor applications, as the effect was also found for PG-1 and anticancer drug doxorubicin against both doxorubicin-sensitive and doxorubicin-resistant human erythromyeloid leukemia cells (K562 cell line).

## Conclusion

The results obtained in current study together with data published by other researchers indicate that synergistic combinations of AMPs with antibiotics as well as with silver nanoparticles are effective tools against multidrug-resistant bacterial strains, including carbapenem-resistant clinical isolates. The most abundant synergy, including the interactions with human endogenous AMPs, is observed for antibiotics targeting protein biosynthesis, such as aminoglycosides and macrolids. Thus, these antibiotics may enhance the antimicrobial activity of host defensive molecules as well as can be used in combinations with AMP-derived antimicrobial drugs.

Our study confirms that the ability of AMPs to permeabilize bacterial membranes plays central role in their synergy with other antimicrobial compounds, but also indicates that this ability could be in turn modulated by the second substance in the combination contributing to the combined effect. Certain cases of synergy with non-membranolytic AMPs suggest that additional mechanisms also exist and require further exploration.

Some cases of increasing cytotoxic activity toward host cells *in vitro* found for AMPs used in combinations with conventional antibiotics point to the importance of further investigation of these effects *in vivo* to avoid them upon a practical application of AMPs against bacteria. However, synergistic action of PG-1 with an antitumor drug doxorubicin indicates the prospect for AMPs in the development of new approaches for combination anticancer therapy.

Taken together our data contribute to the conception of the prospect of an application of antimicrobial peptides of the innate immune systems as non-traditional tools for counteracting drug-resistant bacteria, in particular by their usage in combination with conventional antibiotics.

## Author Contributions

OS and DO contributed conception and design of the study. MZ performed all antimicrobial testing experiments, the statistical analysis of the obtained data, wrote the first draft of the manuscript. DO participated in the experiments on the evaluation of the effects of AMP/antibiotics combination on bacterial membrane permeabilization. TG elaborated doxorubicin-resistant K562 erythroleukemia cell line; IE examined the hemolytic activity of the studied substances, OC carried out MTT tests. OG synthesized and characterized silver nanoparticles. All authors contributed to manuscript revision, read, and approved the submitted version.

### Conflict of Interest Statement

The authors declare that the research was conducted in the absence of any commercial or financial relationships that could be construed as a potential conflict of interest.
