# Supplementary figures and images for: Application of Antimicrobial Peptides of the Innate Immune System in Combination With Conventional Antibiotics—A Novel Way to Combat Antibiotic Resistance?
Source: Front Cell Infect Microbiol. 2019 Apr 30;9:128. doi: 10.3389/fcimb.2019.00128 (PMC6503114; doi:10.3389/fcimb.2019.00128)

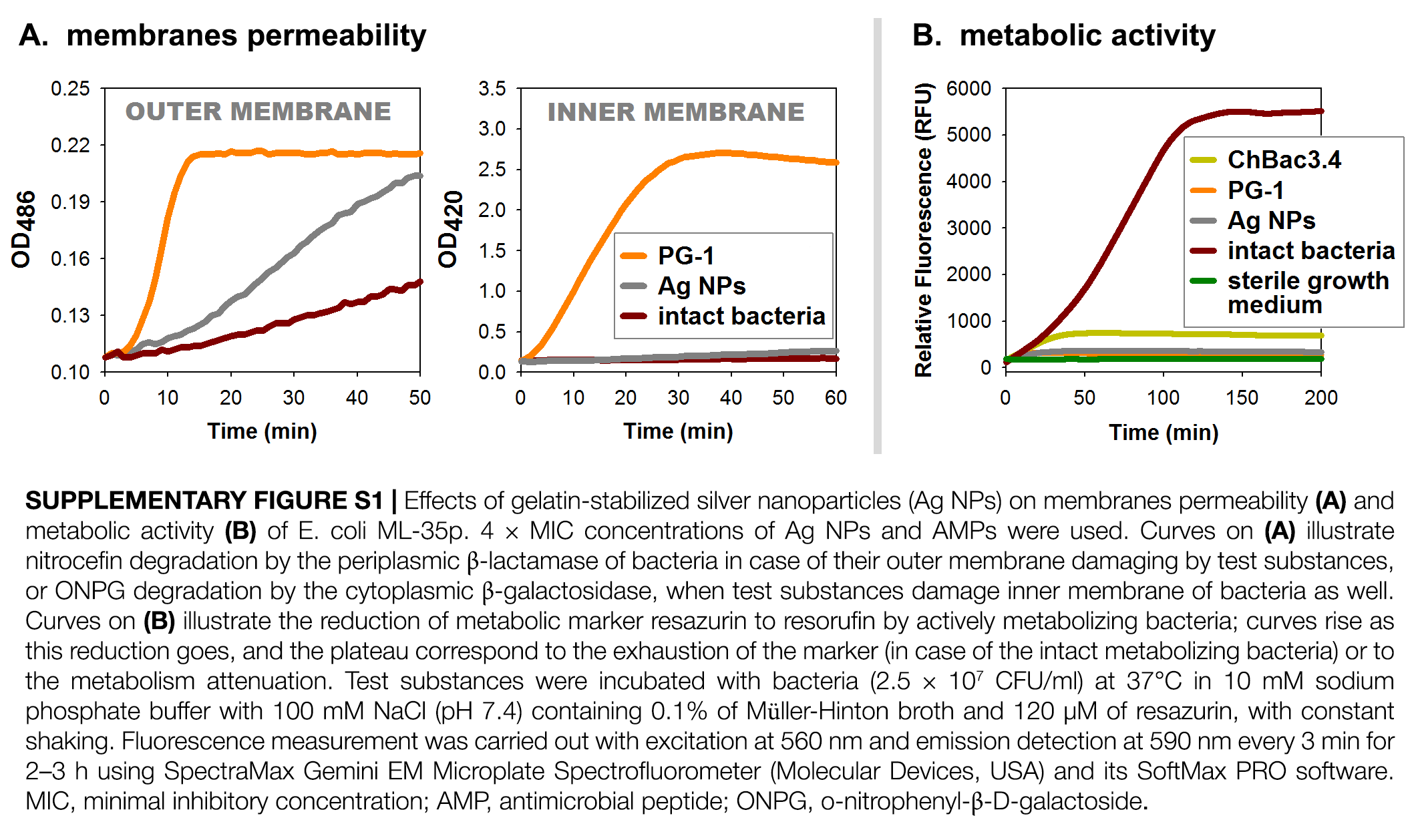

Supplement: Supplementary file 1 [file Image_1.tif]
